# Decreased ATM Function Causes Delayed DNA Repair and Apoptosis in Common Variable Immunodeficiency Disorders

**DOI:** 10.1007/s10875-021-01050-2

**Published:** 2021-05-19

**Authors:** Chantal E. Hargreaves, Silvia Salatino, Sarah C. Sasson, James E. G. Charlesworth, Elizabeth Bateman, Arzoo M. Patel, Consuelo Anzilotti, John Broxholme, Julian C. Knight, Smita Y. Patel

**Affiliations:** 1 Nuffield Department of Medicine and Oxford NIHR Biomedical Research Centre, University of Oxford, Oxford, OX3 9DU UK; 2 Wellcome Centre for Human Genetics, University of Oxford, Oxford, OX3 7BN UK; 3 Oxford University Clinical Academic Graduate School, Medical Sciences Office, John Radcliffe Hospital, University of Oxford, OX3 9DU Oxford, UK; 4 Department of Immunology, Churchill Hospital, Oxford University Hospitals NHS Trust, Oxford, OX3 7LE UK; 5 Clinical Immunology Department, Oxford University Hospitals Trust, Oxford, OX3 9DU UK

**Keywords:** Apoptosis, common variable immunodeficiency disorders, CVID, DNA damage and repair, primary antibody deficiency

## Abstract

**Purpose:**

Common variable immunodeficiency disorders (CVID) is characterized by low/absent serum immunoglobulins and susceptibility to bacterial infection. Patients can develop an infections-only phenotype or a complex disease course with inflammatory, autoimmune, and/or malignant complications. We hypothesized that deficient DNA repair mechanisms may be responsible for the antibody deficiency and susceptibility to inflammation and cancer in some patients.

**Methods:**

Germline variants were identified following targeted sequencing of *n* = 252 genes related to DNA repair in *n* = 38 patients. NanoString nCounter PlexSet assay measured gene expression in *n* = 20 CVID patients and *n* = 7 controls. DNA damage and apoptosis were assessed by flow cytometry in *n* = 34 CVID patients and *n* = 11 controls.

**Results:**

Targeted sequencing supported enrichment of rare genetic variants in genes related to DNA repair pathways with novel and rare likely pathogenic variants identified and an altered gene expression signature that distinguished patients from controls and complex patients from those with an infections-only phenotype. Consistent with this, flow cytometric analyses of lymphocytes following DNA damage revealed a subset of CVID patients whose immune cells have downregulated ATM, impairing the recruitment of other repair factors, delaying repair and promoting apoptosis.

**Conclusion:**

These data suggest that germline genetics and altered gene expression predispose a subset of CVID patients to increased sensitivity to DNA damage and reduced DNA repair capacity.

**Supplementary Information:**

The online version contains supplementary material available at 10.1007/s10875-021-01050-2.

## Introduction

Common variable immunodeficiency disorders (CVID) is the most clinically prevalent cause of primary antibody failure in adults and children, with an incidence of 1:25,000 [1]. Characterized by recurrent infections; low serum levels of IgG, IgM, and/or IgA; and poor-specific antibody responses [2], CVID has a heterogeneous clinical course. The majority of patients have an infections-only phenotype. A subset develops a complex phenotype that can involve autoimmune cytopenia, polyclonal lymphoproliferation, enteropathy, and malignancy [3]. A complex phenotype is associated with a higher mortality rate than other patients and the general population [4].

CVID is a diagnosis of exclusion, with a genetic contribution known for only 10–30% of patients, depending on the cohort [5]. A recent study identified *NFKB1* as the predominant (4%) monogenic cause of a CVID-like phenotype [6]. A genome-wide association study (GWAS) of 363 CVID patients found multiple susceptibility loci, concluding that sporadic CVID is likely a polygenic disease [7]. Our previous whole genome sequencing (WGS) study of 31 sporadic CVID patients confirmed this and identified an enrichment of rare variants in genes related to DNA repair pathways. A majority (54%) of patients had at least one variant in a gene involved in a DNA repair pathway [8]. These data suggest that the accumulation of variants in multiple pathways may contribute to disease pathogenesis.

Double-stranded DNA breaks (DSBs) are potentially dangerous lesions yet are key to an effective immune response through somatic recombination and hypermutation of lymphocyte receptors [9, 10] using multiple DNA repair pathways [11, 12]. DSBs are recognized by ATM, which is activated through its autophosphorylation at serine 1981 following its recruitment by the damage-sensing MRN complex (MRE11A-Rad50-NBS1) [13]. The histone H2A variant, H2AX, is phosphorylated at serine 139 following DNA damage, by ATM, ATR, and DNA-PKcs to become γH2AX [14]. Foci of γH2AX spread at the sites of DSBs and initiate the recruitment and maintenance of other factors for their repair [15].

V(D)J recombination involves programmed DSBs repaired by non-homologous end-joining and homologous recombination repair. The mismatch and base excision repair pathways generate and repair the base mismatches and DNA breaks essential for somatic hypermutation and class-switch recombination [16, 17]. The essential role of these pathways in the immune system is best demonstrated by primary immune deficiencies caused by monogenic defects in DNA repair genes [18]. There are multiple lines of evidence for the role of defective DNA repair in CVID given the low serum immunoglobulins, defective isotype switching, alterations in somatic hypermutation [19, 20] and increased risk of malignancy compared to the general population [3, 21].

We hypothesized that multiple variants in genes related to DNA repair pathways predispose to CVID and its associated complications. Here, we sought to validate our WGS data through targeted resequencing of genes related to DNA repair in an extended CVID cohort and functional testing of the DNA repair response in vitro by measuring repair markers and damage-induced apoptosis.

## Methods

### Characteristics of Patient and Control Cohorts

Patients with CVID (*n* = 38) or primary antibody deficiency (PAD; *n* = 2) were recruited through the Clinical Immunology Department, John Radcliffe Hospital, Oxford, UK. The median age of patients at the time of study sample collection was 50.1 years (range 20.8–80), and 50% were female. The median age was 49.3 years for infections-only patients and 51 years for complex patients. A monogenic cause was excluded by WGS in 19 CVID patients. PAD patients did not meet CVID diagnostic criteria, as onset was < 4 years old, and were included in case dysregulated DNA repair was a cause of their antibody failure. Patients met the European Society for Immunodeficiencies diagnostic criteria at the time of enrolment [2]. All participants gave informed written consent, and studies were performed according to the Declaration of Helsinki with South Central Research Ethics Committee approval (12/SC/0044). Control blood samples (*n* = 13) were obtained from self-reporting healthy staff members at the University of Oxford through the Oxford Gastrointestinal Illness Biobank (16/YH/0247). The median age of controls was 35 years (range 24–48), and 64% were female. Patients’ clinical characteristics are described in Table S1**.** Due to limitations on primary cellular material, it was not possible to perform all experiments on all participants (Table S2).

### HaloPlex HS Library Preparation, Sequencing, and Analysis

A custom HaloPlex HS targeted enrichment assay (Agilent) of 252 genes related to DNA repair was designed using Agilent’s Sure Design software. Custom probes were designed targeting the exons and 5′ and 3′ untranslated regions with an extra 50 base pairs (bp) using the hg19 genome build. Probes were also targeted to 23 genomic regions to improve probe coverage (Table S3).

HaloPlex HS libraries were prepared for *n* = 36 CVID and *n* = 2 PAD patients. Genomic DNA was extracted from peripheral blood mononuclear cells (PBMCs) using FlexiGene DNA or DNeasy Blood and Tissue kits (Qiagen). A 50 ng of DNA was enzymatically digested and hybridized for 16 h to custom probes. Circularized fragments were ligated, captured, and amplified by PCR. Libraries were purified with AMPure beads (Beckman Coulter) and quantified with a Bioanalyzer 2100 and High Sensitivity DNA chips (Agilent). Equimolar library pools were prepared for 150 bp paired-end sequencing with an Illumina HiSeq 4000 (Illumina) at Oxford Genomics Centre, Wellcome Centre for Human Genetics, University of Oxford.

### Bioinformatic Analysis Pipeline

Quality control for the sequenced reads was performed with an in-house Python-based pipeline. Prior to alignment, Agilent SurecallTrimmer (version 4.0.1) was used to trim adapter sequences and low-quality bases and mask enzyme footprints. Reads were mapped to the hs37d5 version of the human reference build 37 using BWA mem (version 0.7.15) [22], and only reads in proper pairs and with insert sizes ranging from 30 to 625 were retained using bamtools (https://github.com/pezmaster31/bamtools). UMIs were added using AddUMIsToBam (https://github.com/mbusby/AddUMIsToBam). Picard (version 2.9.2) http://broadinstitute.github.io/picard/) was used to fix mate CIGAR strings, merge files from multiple sequencing lanes, and deduplicate BAM files.

Platypus (version 0.8.1, default parameters) [23] identified SNVs and short (< 50 bp) indels within each sample. Variants were annotated with an in-house pipeline written in Python using the Variant Effect Predictor framework (VEP, version 77) [24] as well as 1000 Genomes Project [25], UK10K [26], Exome Variant Server (ESP6500, http://evs.gs.washington.edu/EVS/), and Exome Aggregation Consortium (ExAC, http://exac.broadinstitute.org/) databases. Known associations with diseases were screened using HGMD (http://www.hgmd.cf.ac.uk) and ClinVar [27].

Variants were filtered using BrowseVCF [28] based on the criteria outlined in Table S4. Oncoplots were generated using the Maftools R Bioconductor package [29]. Variants were prioritized based on being novel and/or predicted pathogenic using American College of Medical Genetics and Genomics (ACMG) guidelines [30] (Table 1). Variants present in public databases with an unknown significance or predicted benign/likely benign are shown in Table S5.

**Table 1:** Summary of novel, pathogenic and likely pathogenic variants

Gene	dbSNP ID	Location	Reference allele	Transcript variant	Protein variant	Consequence	ACMG	SIFT	PolyPhen	CADD	Mutation assessor	FATHMM	M-CAP	GERP + +	1000 Genomes	ExAC	gnomAD	Patients
*MRE11A*	rs745677716	11:94,178,974		c.1783 + 1411 T > C		Splice donor	Pathogenic	-	-	-	-	-	-	-	-	0.001	0.002	PID002
*USP45*	rs554927779	6:99,916,419	T	c.1008delA	p.V337fs*9	Frameshift	Pathogenic	-	-	-	-	-	-	-	0.1	0.3	0.19	C099
*BRCA1*		17:41,249,292	C	c.562G > T	p.E188*	Stop gained	Likely pathogenic	-	-	38	-	-	-	5.49	-	-	-	PID019
*TOP3A*		17:18,205,719	CA	c.671_672delTG	p.L224fs*8	Frameshift	Likely pathogenic	-	-	-	-	-	-	-	-	-	-	PID016
*CHD3*		17:7,793,884		c.391-131delA		Splice region	Uncertain significance	-	-	-	-	-	-	-	-	-	-	D438
*POLQ*		3:121,206,144		c.5629 + 5G > A		Splice region	Uncertain significance	-	-	-	-	-	-	-	-	-	-	PID046
*REV3L*		6:111,702,623	CTTCCA	c.1115_1120delTGGAAG	p.V372_E373del	Inframe deletion	Uncertain significance	-	-	-	-	-	-	-	-	-	-	PID099
*TP53BP1*		15:43,720,207		c.3828 + 7G > T		Splice region	Uncertain significance	-	-	-	-	-	-	-	-	-	-	C192; PID018; PID034; PID068
*POLR2D*		2:128,615,616	A	c.59 T > C	p.L20P	Missense	Uncertain significance	D	PrD	28	M	-	D	4.09	-	-	-	PID064
*POLR2B*		4:57,876,929		c.1339C > T	p.L522F	Missense	Uncertain significance	D	PrD	31	H	D	D	5.64	-	-	-	C099; PID005; PID008; PID018; PID020; PID068
*DDB1*		11:61,081,648	C	c.1624G > T	p.D542Y	Missense	Uncertain significance	D	PrD	32	M	T	D	5.84	-	-	-	PID027
PRKDC*		8:48,805,824	A	c.3722 T > C	p.L1241P	Missense	Uncertain significance	D	PrD	25	-	T	D	5.82	-	-	-	PID011
*CHD8*		14:21,853,930	G	c.7588C > G	p.R2530G	Missense	Uncertain significance	D	PoD	24	N	D	D	5.26	-	-	-	C192
*MRE11A*		11:94,180,422	C	c.1746G > T	p.Q582H	Missense	Uncertain significance	D	PoD	25	M	T	D	6.08	-	-	-	PID017; PID056
*PMS1*		2:190,682,823	G	c.499G > A	p.D167N	Missense	Uncertain significance	D	B	25	L		D	5.36	-	-	-	PID064
*CHD8*		14:21,897,160	C	c.1178G > A	p.R393K	Missense	Uncertain significance	D	B	23	L	D	D	5.44	-	-	-	PID019
AICDA*		12:8,759,495	G	c.122C > T	p.S41F	Missense	Uncertain significance	T	PrD	25	M	T	D	5.36	-	-	-	PID031
*MRE11A*		11:94,180,427	C	c.1741G > C	p.G581R	Missense	Uncertain significance	T	PrD	26	M	T	D	6.08	-	-	-	PID017; PID056
*EME1*		17:48,457,732	T	c.1406 T > G	p.V469G	Missense	Uncertain significance	T	PoD	23	M	T	D	5.9	-	-	-	PID017
*ERCC4*		16:14,014,072	A	c.50A > G	p.E17G	Missense	Uncertain significance	T	PoD	26	M	T	D	5.13	-	-	-	PID064
CHD7*		8:61,775,197	A	c.8062A > G	p.I2688V	Missense	Uncertain significance	T	PoD	24	N	T	T	5.59	-	-	-	PID006
PRKDC*		8:48,706,876	G	c.10645C > A	p.H3549N	Missense	Uncertain significance	T	B	22	M	-	T	5.74	-	-	-	PID083
TREX1*		3:48,508,908	A	c.854A > G	p.E285G	Missense	Uncertain significance	T	B	14	L		T	4.5	-	-	-	C099
*CHEK2*		22:29,121,049	C	c.508G > T	p.V170L	Missense	Uncertain significance	T	B	24	L	D	D	5.87	-	-	-	PID027
*MSH4*		1:76,363,751	G	c.2515G > A	p.E839K	Missense	Uncertain significance	T	B	24	N	D	T	5.66	-	-	-	PID033
LIG1*		19:48,624,537	G	c.2275C > G	p.L759V	Missense	Uncertain significance	T	B	23	L	T	D	5.29	-	-	-	PID018
*TOP2A*		17:38,562,923	C	c.1756G > A	p.E586K	Missense	Uncertain significance	T	B	22	L	T	D	5.51	-	-	-	PID006; PID033
*RAD17*		5:68,680,732	G	c.650G > A	p.R206K	Missense	Uncertain significance	T	B	15	N	T	T	5.22	-	-	-	C166
*RAD17*		5:68,680,735	C	c.653C > A	p.T207N	Missense	Uncertain significance	T	B	22	L	T	T	5.22	-	-	-	PID049
*REV3L*		6:111,737,595	T	c.220A > G	p.S74G	Missense	Uncertain significance	T	B	20	L	T	T	5.31	-	-	-	PID018

^*^Denotes known PID gene. *B* benign, *D* damaging, *H* high, *L* low, *M* medium, *N* neutral, *PoD* possibly damaging, *PrD* probably damaging, *T* tolerated

### PBMC Isolation

PBMCs were isolated with Lymphoprep (Stem Cell Technologies), washed, and stored in fetal bovine serum (FBS; Sigma) with 10% DMSO (Sigma) in liquid nitrogen. Defrosted cells were washed twice and re-suspended in RPMI‐1640 (Lonza) supplemented with 10% FBS, sodium pyruvate, non-essential amino acids, β-mercaptoethanol, penicillin, and streptomycin (Sigma). Viability and cell counts were assessed using a hemocytometer and trypan blue.

### Targeted mRNA Gene Expression Profiling

PBMCs (0.5 × 10^6^) from *n* = 7 controls and *n* = 20 patients were pelleted at 1600 rpm for 8 min, lysed with RLT buffer (Qiagen), diluted 1:3 with deionized water, and stored at -80 °C until analysis. Cell lysates (80,000 cells) were hybridized with NanoString nCounter DNA Damage Repair PlexSet-96 probes and 0.2 mg/mL Proteinase K (Beckman Coulter) for 17.5 h at 67 °C. Libraries were applied to an nCounter SPRINT Cartridge and nCounter SPRINT Profiler (NanoString). Raw data were analyzed using nProfiler software (NanoString), DESeq2 [31] and RStudio (version 1.2.5019). Differentially expressed genes were classified as having a log^2^ FoldChange > 0.5 and a false discovery rate (FDR) < 0.05.

### Flow Cytometry

PBMCs from *n* = 11 controls, *n* = 32 CVID and *n* = 2 PAD patients were either untreated or exposed to 5 Gy of γ-irradiation and cultured for 1 or 24 h. Cells were stained with Fixable Live/Dead eF780 (ThermoFisher), CD3-BV785 (clone: SK7), CD4-BV510 (SK3), CD8-PE-Cy7 (HIT8a) and CD19-BV605 (HIB19) in PBS, 5% bovine serum albumin, and 0.09% sodium azide for 10 min at room temperature. Cells were washed and fixed with eBioscience FoxP_3_/Transcription Factor Staining Buffer Set fixative (eBioscience) for 1 h at 4 °C. Cells were permeabilized in the presence of γH2AX^Ser139^-FITC (2F3), ATM^Ser1981^-PE (10H11.E12), 53BP1-AF700 (cat. no. NB100-904; Novus Biologics) and cleaved PARP^Asp214^-PE-CF594 (F21-852, BD Biosciences) for 1 h at 4 °C. Antibodies were obtained from Biolegend unless otherwise stated. Samples were analyzed using an LSR Fortessa (BD Biosciences) and FlowJo version 9 software (Tree Star).

### Statistical Analyses

Statistical analyses were performed using GraphPad Prism version 8. Non-parametric distribution of data was determined using D’Agostino and Pearson test. Statistical significance is defined as *p < 0.05, **p < 0.01, ***p < 0.001, and ****p < 0.0001.

## Results

### Targeted Resequencing of DNA Repair-Related Genes in CVID

Our previously published WGS analysis provided evidence for the polygenic nature of sporadic CVID and a disease etiology based on the contribution of multiple interacting genes and pathways [8]. Of particular interest was the enrichment of likely pathogenic rare variants in genes related to DNA repair pathways. We sought to validate these data through targeted resequencing of a custom selection of 252 genes related to DNA repair pathways in a cohort of 36 sporadic CVID and 2 PAD patients.

**Fig. 1:**
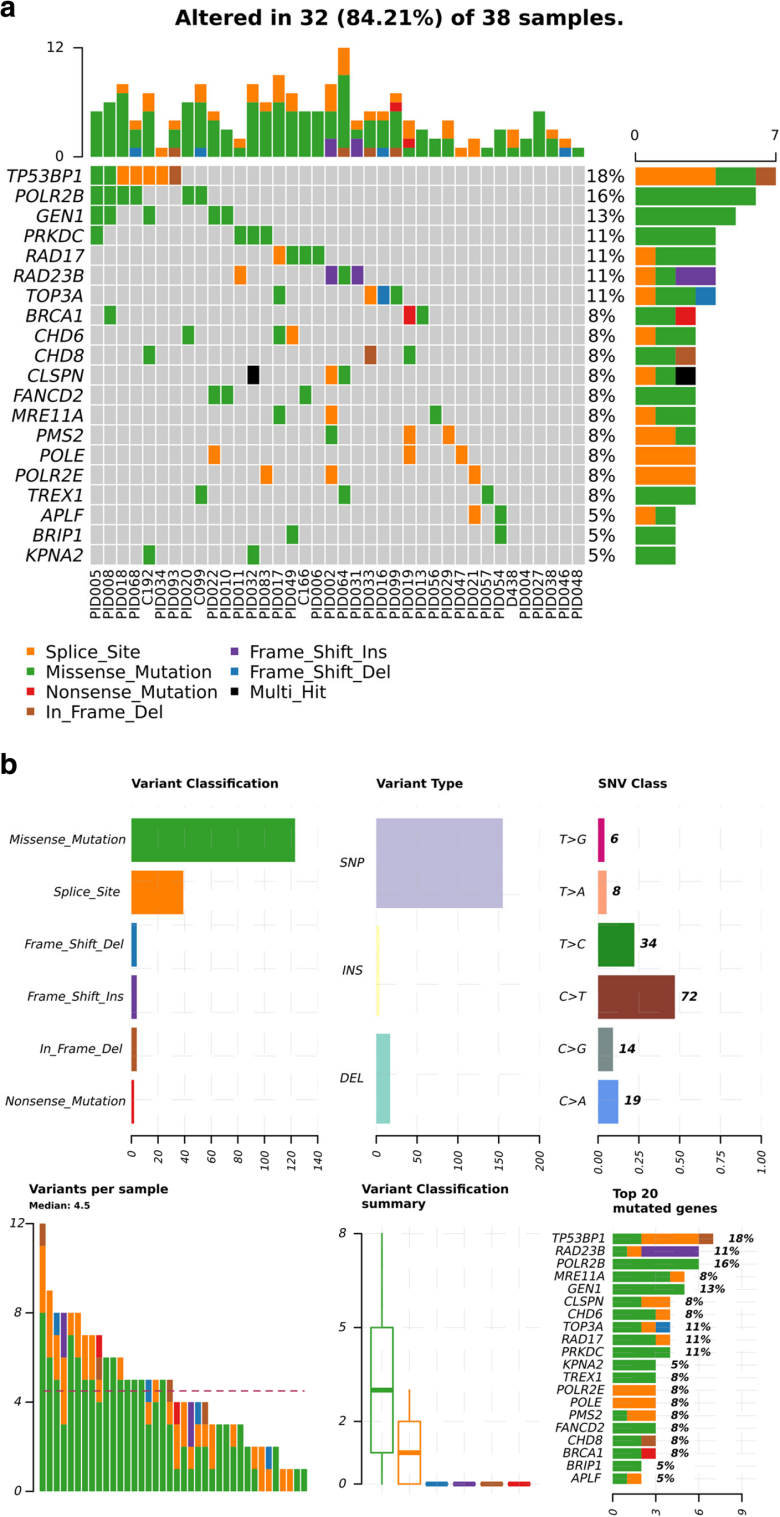
CVID patients have rare germline variants in genes related to DNA repair pathways. Classification across the cohort of (**a**) variant classification, variant type, single nucleotide variant class (transition or transversion), the number of variants per sample, variant classification summary and the top 20 frequently mutated genes. In the oncoplot (**b**), each row represents a gene, and each column a patient. The histogram summarises the number and type of variant per sample. In each row, the type of variant in a given gene is colour-coded with the % variability of that gene across the cohort

Targeted DNA resequencing of *n* = 38 patients identified a total of 84,922 variants. Filtering excluded variants with a > 1% frequency in 1000 genomes [25], ExAC [32], ESP6500, and UK10K [26] public databases (Table S4), to allow identification of causative and disease-modifying variants. Exclusion of synonymous, non-coding, and low complexity region variants left 136 heterozygous variants, of which 32 were novel and 24 were below 0.1% frequency. The majority of the polymorphisms were missense, SNVs were more prevalent than insertions or deletions, and C > T transitions were the most common SNV type. There was a median of 4.5 variants per individual (range 1–12), a median of 4 (range 2–12) among complex patients and a median of 5 (range 1–9) among infections-only patients. The most variable genes were *TP53BP1* (18%), *POLR2B* (16%), and *GEN1* (13%) (Fig. 1a). Each patient’s combination of variable genes and variant types is summarized by oncoplot in Fig. 1b.

We identified predicted pathogenic variants in *MRE11A* (splice donor c.1783 + 1411 T > C) and *USP45* (p.V337fs*9) and likely pathogenic variants in *BRCA1* (p.E188*) and *TOP3A* (p.L224fs*8). Novel genes classified as of uncertain significance based on their absence from public databases are detailed in Table 1.

For genes involved in V(D)J recombination, we found novel missense variants in *AICDA* (p.S41F) and *PRKDC* (p.L1241P) (Table 1) and rare missense variants in *DCLRE1C* (p.L214M), DNTT (p.R460Q), *PRKDC* (p.R2595H), *POLL* (p.G376S and p.303S), and *POLM* (p.G220A) (Table S5). We identified novel and rare variants in pathways including homology-directed recombination (*MRE11A* p.Q582H, p.G581R, and c.1867 + 2 T > C), mismatch repair (*MSH3* p.Glu437fs*10), nucleotide excision repair (*ERCC4* p.E17G), and chromatin organization (*CHD7* p.S842Y and *CHD8* p.R293K). Variants in these genes could cause antibody deficiency through inefficient V(D)J recombination, somatic hypermutation, and/or class-switch recombination. We did not find an association with variable genes and CVID phenotype (Fig. S1).

Every patient shared a variable gene with at least one other patient but no more than two variants with another (Table 1, Table S5). The most frequent variant in the cohort, *POLR2B* p.L522F, was found in six patients. PID017 and PID056 shared the two novel variants in *MRE11A*, p.Q582H, and p.G581R (Table 1).

### Altered Expression of Genes Related to DNA Repair in Complex CVID Patients

**Fig. 2:**
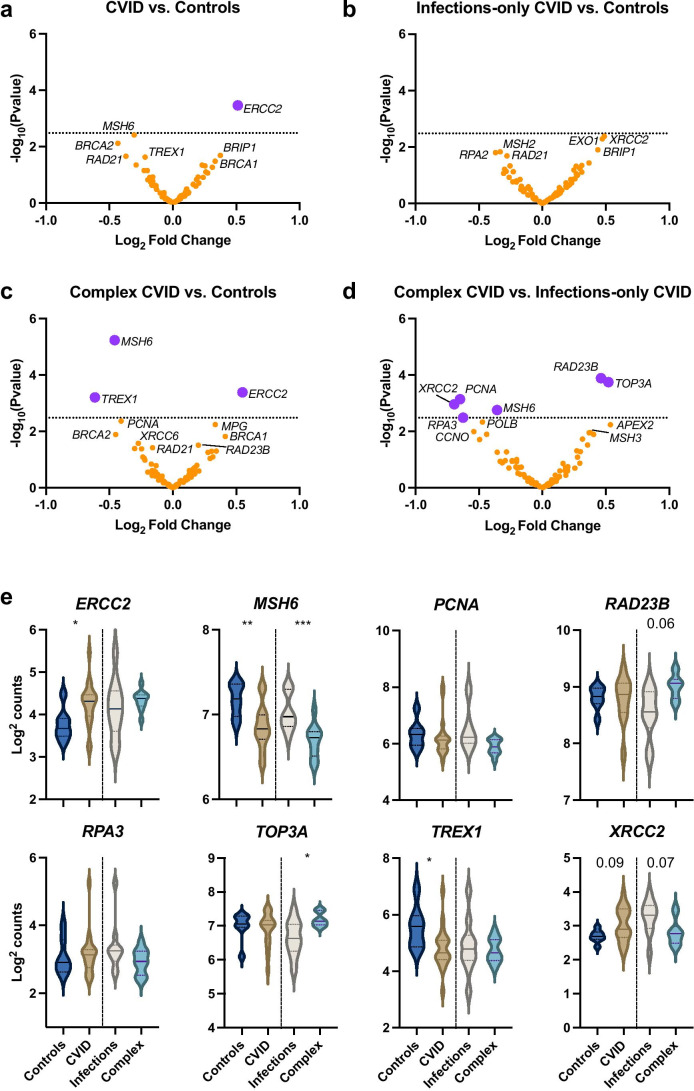
Differential gene expression of genes related to DNA repair pathways is altered in complex patients versus controls and infections-only patients. Volcano plots summarising the differential gene expression between (**a**) n=20 patients and n=7 controls, (**b**) n=10 infection-only phenotype versus controls, (**c**) n=10 complex phenotype patients versus controls and (**d**) complex phenotype patients versus infections-only patients. Genes whose expression is significantly different are shown in purple. The dotted line represents a false discovery rate <0.05. (**e**) Key genes and their log^2^ counts are summarised in controls, patients and their phenotypic subgroups. Statistical significance is measured by Mann-Whitney test

We assessed the mRNA expression of 90 genes related to DNA repair in PBMC lysates (Fig. 2a–e).

Expression of *ERCC2* was higher in CVID patients versus controls (Fig. 2a) and in complex versus infections-only patients (Fig. 2c**)**. We did not find statistically significant differences in gene expression between infections-only CVID patients and controls (Fig. 2b). In complex patients, compared to controls, we found decreased expression of *MSH6* and *TREX1* (Fig. 2c, e) and compared to infections-only patients, increased expression of *TOP3A* and *RAD23B* and decreased expression of *PCNA*, *RPA3*, *XRCC2*, and *MSH6* (Fig. 2d). The associations with *RPA3* and *PCNA* appear due to their elevated expression in one or two patient samples, rather than in the cohort as a whole (Fig. 2e).

Alterations in gene expression in PBMCs could be a result of changes in cellular subfraction frequencies. We did not detect significant differences in the frequencies of CD19^+^ B cells, T cells (CD3^+^, CD4^+^, and CD8^+^), and CD16^+^CD56^+^ NK cells, as measured by TBNK assay, between CVID phenotypic sub-groups (Fig. S2), and few were outside reference ranges (Fig. S3). There was a positive association between *ERCC2* log^2^ counts and NK cell frequencies (*r* = 0.58, *p* = 0.009) and a negative correlation with CD3^+^ T cell frequencies (*r* = -0.51, *p* = 0.03). *TREX1* expression was positively correlated with CD4^+^ T cell frequencies (*r* = 0.6, *p* = 0.007) and negatively correlated with CD8^+^ T cell frequencies (*r* = -0.54, *p* = 0.02). We did not observe a significant correlation with the other genes examined (Fig. S3).

### CVID Patients Have Increased Levels of γH2AX Compared to Controls

We measured γH2AX kinetics in PBMCs from controls and patients by flow cytometry. A representative gating strategy of immune cell subsets is shown in Fig. S4.

Patients had higher baseline frequencies of γH2AX^+^ cells than controls following 1 h in culture in CD4^+^ T cells (*p* = 0.0002), CD8^+^ T cells (*p* ≤ 0.0001), and CD19^+^ B cells (*p* ≤ 0.0001) and at 24 h in CD4^+^ T cells (*p* ≤ 0.0001), CD8^+^ T cells (*p* = 0.0005), and CD19^+^ B cells (*p* = 0.0034) (Fig. S5).

**Fig. 3:**
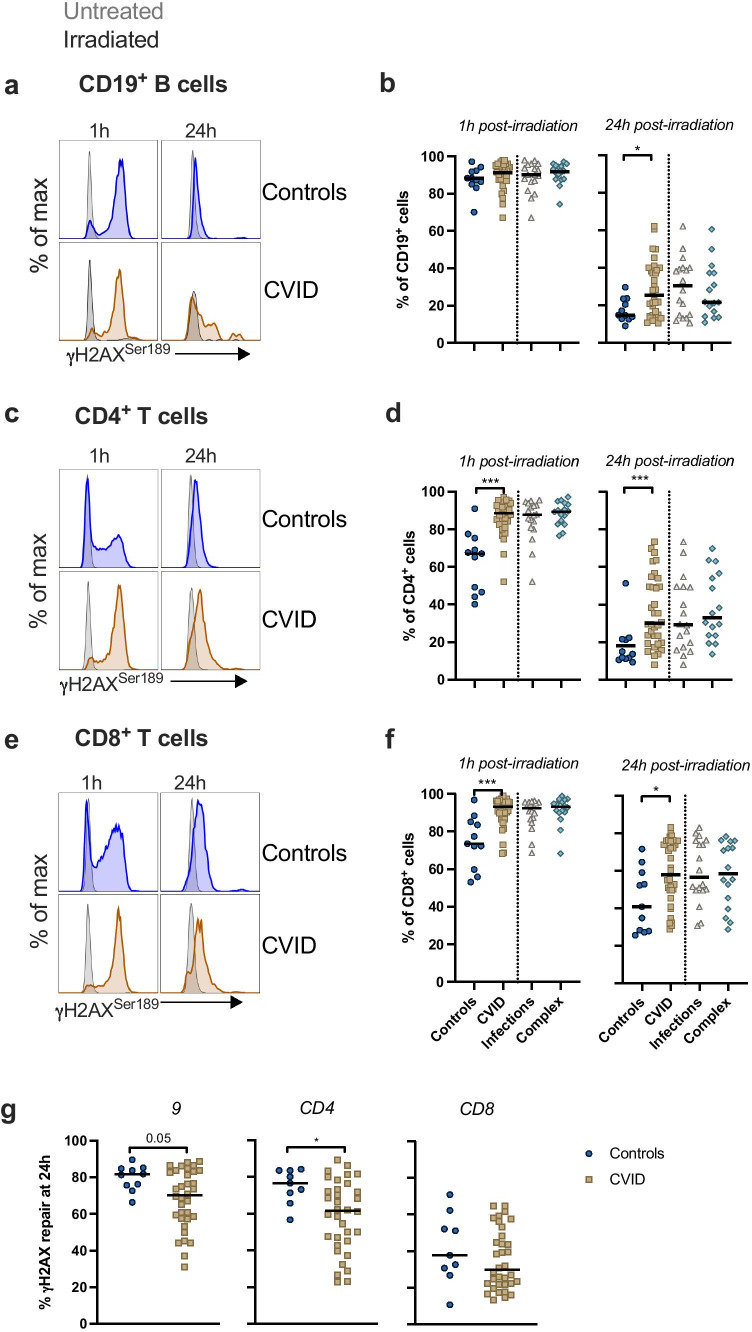
CVID B and T cells have higher levels of the DDR marker, γH2AX, than controls. PBMCs from controls (n=11) and CVID and PAD patients (n=34 and n=2, respectively) were analysed by flow cytometry. **a**,** c**,** e** Representative histograms of intra-nuclear gH2AX expression after 1 and 24h of culture in CD19^+^ B cells, CD4^+^ T cells and CD8^+^ T cells. Untreated cells are shown in grey and cells exposed to 5 Gy g-irradiation in a control (blue) and CVID patient (gold). The frequency of gH2AX^+^ cells in **b** CD19^+^ B cells, **c** CD4^+^ T cells and **d** CD8^+^ T cells 1 and 24h post-irradiation. Displayed are controls (circles), CVID patients (squares), CVID patients with an infections-only phenotype (triangles, n=19) and CVID patients with a complex phenotype (diamonds, n=15). **e** The % of DNA repair presented as the change in gH2AX^+^ cell frequency from 1 to 24h post-irradiation. Each symbol represents an individual control or patient. Bar represents the median. Statistical significance was determined by Mann-Whitney test

Among CD19^+^ B cells, we observed a higher frequency of γH2AX^+^ cells at 24 h in patients versus controls (*p* = 0.044), but not at 1 h post-irradiation (Fig. 3a, b). For CD4^+^ T cells, patient cells displayed increased frequencies of γH2AX^+^ cells compared to controls 1 h (*p* ≤ 0.0001) and 24 h post-irradiation (*p* ≤ 0.0001) (Fig. 3c, d). Similarly, for CD8^+^ T cells, 1 h (*p* = 0.0004) and 24 h post-irradiation (*p* = 0.04) (Fig. 3e, f). No difference was observed between patient phenotypic sub-groups for any of the lymphocyte subsets examined. Nor was increased γH2AX observed for the PAD patients. To assess DNA repair capacity, we measured the percent change from 1- to 24-h post-damage. Repair was significantly delayed in CVID CD4^+^ (*p* = 0.02) and CD19^+^ (*p* = 0.05) cells. There was no significant delay in the clearance of CD8^+^γH2AX^+^ cells (*p* = 0.31), likely due to heterogeneity among control cells (Fig. 3g).

An accumulation of DNA damage is reported in aging memory T cells over naïve cells due to the increasing presence of terminally differentiated, senescent, and chronically antigen-activated T cells [33–35]. At 24 h post-irradiation, we observed a positive association with T cell γH2AX levels and increasing age of participants (Fig. S6) but not the other markers and cell types.

### A Subset of CVID Patients’ B Cells Downregulates pATM 24 h Post-DNA Damage

**Fig. 4:**
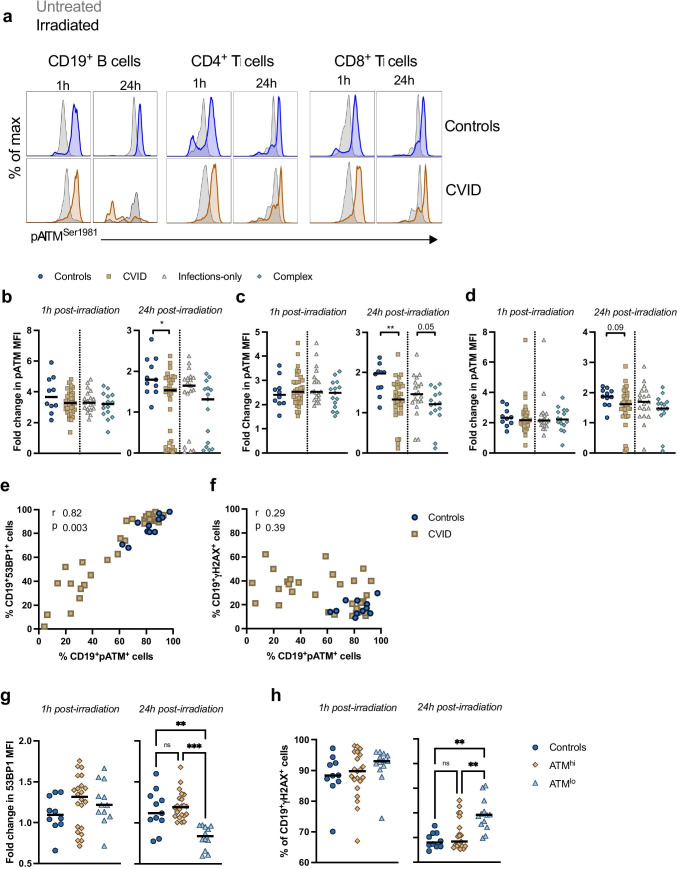
A subset of CVID patients’ B cells downregulate pATM 24 h after DNA damage. (**a**) Representative histograms of intra-nuclear pATM expression after 1 and 24h of culture in CD19^+^ B cells, CD4^+^ T cells and CD8^+^ T cells. Untreated cells are shown in grey and cells exposed to 5 Gy g-irradiation in a control and CVID patient in bold. The fold change in median fluorescence over untreated cells is shown for (**b**) CD19^+^ B cells, (**c**) CD4^+^ T cells and (**d**) CD8^+^ T cells 1 and 24h post-irradiation. Displayed are controls (circles, n=11), CVID and PAD patients (squares, n=34), CVID patients with an infections-only phenotype (triangles, n=19) and CVID patients with a complex phenotype (diamonds, n=15). Bar represents the median. Statistical significance was determined by Mann-Whitney test. Spearman rank correlations of the frequency of CD19^+^pATM^+^ cells and frequencies of (**e**) CD19^+^53BP1^+^ cells and (f) CD19^+^gH2AX^+^ cells. CVID samples were classified as ATM^hi^ or ATM^lo^ based on their B cell pATM expression 24h post-irradiation. For B cells, the (**g**) fold change of 53BP1 over untreated and (**h**) frequency of gH2AX^+^ cells were compared amongst controls (circles), ATM^hi^ (diamonds, n=21) and ATM^lo^ (triangles, n=13) groups. Statistical significance was determined by two-way ANOVA with Tukey’s test for multiple comparisons. Each symbol represents an individual control or patient.

Activation of ATM is key to orchestrating DDR responses, including phosphorylating downstream effectors, regulation of the cell cycle, and initiating apoptosis [36]. We measured pATM levels among lymphocyte subsets by flow cytometry (Fig. 4a). At baseline (Fig. S5), B cells had decreased pATM compared to controls (*p* ≤ 0.001), likely due to apoptosis following time in culture. We did not observe differences in pATM expression in CD19^+^ B cells, CD4^+^ T cells or CD8 + T cells between patients and controls or patient sub-groups 1 h post-irradiation (Fig. 4b–d). However, 24 h post-DNA damage, there was a significant downregulation in pATM in CVID B cells (*p* = 0.03), CD4^+^ T cells (*p* = 0.003), and a trend in CD8^+^ T cells (*p* = 0.09) compared to controls (Fig. 4b–d). There was a trend to a greater decrease in pATM in complex CVID patient CD4^+^ T cells compared to infections-only cells (*p* = 0.05; Fig. 4c). The decrease in CVID B cell pATM levels was driven by *n* = 12/38 patients, five with an infections-only phenotype and seven a complex phenotype.

The p53-binding protein 1 (53BP1), a crucial component of DSB repair downstream of ATM, influences cells’ choice of the NHEJ pathway [37]. There was increased CD4^+^ cell expression of 53BP1 in CVID patients compared to controls 1 h following irradiation (*p* = 0.007) and a trend for CD8^+^ cells (*p* = 0.06) (Fig. S7). There was decreased expression at baseline in CVID patient cells (CD19^+^ *p* = 0.02, CD4^+^ *p* = 0.009, and CD8^+^ *p* = 0.003) compared to controls at 1 h (Fig. S5).

There was a positive correlation between the frequency of CD19^+^pATM^+^ cells and CD19^+^53BP1^+^ cells (*r* = 0.82, *p* = 0.003) b ut not with CD19^+^γH2AX^+^ cells (*r* = 0.29, *p* = 0.39) (Fig. 4e–f). We designated patients as pATM^hi^ if their B cells had > 50% ATM^+^ cells and as pATM^lo^ if samples had < 50% pATM^+^ cells 24 h post-irradiation. At 24 h, compared to controls and pATM^hi^ cells, the pATM^lo^ group had decreased 53BP1 (*p* = 0.002 and *p* ≤ 0.001, respectively) (Fig. 4g) and increased γH2AX^+^ cells (both *p* ≤ 0.001) (Fig. 4h).

To determine whether the ATM^lo^ patient groups were genetically distinct from the ATM^hi^ group, we compared the variable genes between both groups. There was *n* = 4 shared genes, *n* = 48 and *n* = 14 unique to ATM^hi^ and ATM^lo^ groups, respectively (Fig. S8a), and a median of 2.2 variants in the ATM^hi^ group and 1.2 in the ATM^lo^ group. There was no significant difference in *ATM* expression*,* but there was a trend of higher expression of *LIG4* and *RAD23B* in ATM^lo^ samples (both *p* = 0.06) (Fig. S8b). However, significance was lost upon correction for multiple testing.

### Immune Cells from a Subset CVID Patients Have Increased Spontaneous and DNA Damage-Induced Apoptosis

Given the mutagenic potential of unresolved DNA lesions, severe, or unrepaired DNA damage should trigger apoptosis [38]. We measured caspase-3′s substrate, poly (ADP-ribose) polymerase (PARP). Cleaved PARP helps cells maintain viability, and its cleavage by caspase-3 is considered a hallmark of apoptosis [39, 40].

**Fig. 5:**
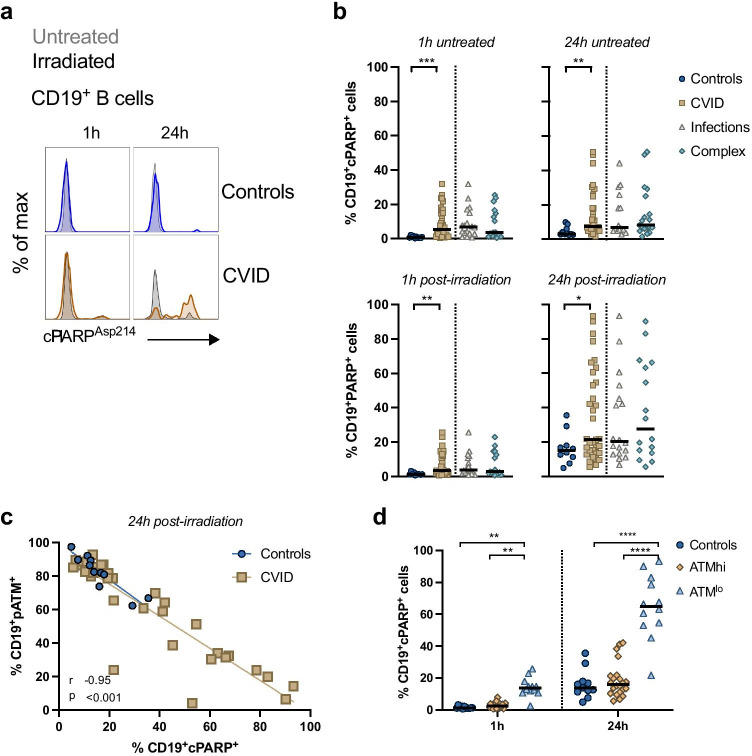
CVID B cells display increased apoptosis which is inversely correlated with pATM levels. **a** Representative gating of early apoptotic marker, cPARP^+^, after 1 and 24h of culture in CD19^+^ B cells. Untreated cells are shown in grey and cells exposed to 5 Gy g-irradiation in a control and CVID patient in bold. **b** Frequency of CD19^+^cPARP^+^ cells in (circles, n=11), CVID and PAD patients (squares, n=34), CVID patients with an infections-only phenotype (triangles, n=19) and CVID patients with a complex phenotype diamonds, n=15). Statistical significance was determined by Mann-Whitney test. **c** Spearman rank correlations of pATM^+^ cells and cPARP^+^ cells amongst CD19^+^ B cells. Each symbol represents an individual control or patient. **d** Frequency of cPARP^+^ cells following irradiation in n=11 controls (circles), ATM^hi^ (diamonds, n=22) and ATM^lo^ (triangles, n=12) patient groups. Statistical significance was determined by two-way ANOVA with Tukey’s test for multiple comparisons. Each symbol represents an individual control or patient. Bars represents the median.

CVID T cells displayed increased basal and DNA damage-induced apoptosis, compared to controls (Fig. S9). We found increased spontaneous cleaved PARP expression in CVID patient B cells compared to controls at 1 h and 24 h post-irradiation (*p* ≤ 0.00 and *p* = 0.002, respectively) and following irradiation (*p* = 0.002 and *p* = 0.04, respectively) (Fig. 5a, b). This was particularly evident 24 h post-DNA damage induction, where a sub-group of CVID patients had significantly increased apoptosis, suggesting a role for the DNA damage response (Fig. 5b).

The frequencies of cleaved PARP^+^ cells were negatively correlated with the frequency of ATM^+^ cells (*r* = -0.95, *p* ≤ 0.001) (Fig. 5c). We analyzed the frequencies of cleaved PARP^+^ B cells among ATM^lo^ and ATM^hi^ groups. The ATM^lo^ group had higher frequencies of cleaved PARP^+^ cells compared to controls at 1 h (*p* = 0.0074) and 24 h (*p* ≤ 0.0001) and ATM^hi^ cells at 1 h (*p* = 0.0044) and 24 h (*p* ≤ 0.0001) post-irradiation (Fig. 5d).

## Discussion

While low or absent immunoglobulins are a hallmark feature of CVID and predispose to recurrent infections, patients are also at greater risk of malignancy than the general population [21, 41]. In vitro radiosensitivity was first reported in CVID in the 1990s [42]. Polymorphisms in genes related to mismatch repair have been found at higher frequencies in an adult CVID cohort and a selective IgA deficiency cohort compared to controls and resulted in increased radiosensitivity in vitro in patient-derived and transfected cell lines [43]. Our WGS study found rare and novel variants in genes involved in V(D)J recombination and DNA repair [8]. We hypothesized that multiple heterozygous variants in DNA repair genes, which individually would have a small effect size, combine in distinct or overlapping pathways to predispose to sporadic CVID and its complications. We applied a targeted approach to understanding the genetic and cellular responses of DNA repair in CVID.

The most frequently variable gene, *TP53BP1*, was found to have four variants in seven patients. Minimal sharing of variable genes was observed between patients, fitting a polygenic model. As in Offer et al. [43], our cohort included variants in *MLH3*, *MSH3*, and *MRE11A*. We identified 15 novel variants in known PID genes: *AICDA*, *ATM*, *DCLRE1C*, *CHD7*, *PRKDC*, *RAG1*, and *RNF168*. A GWAS of CVID found that many of the identified variants were unique to individual patients [7] and recent whole exome sequencing uncovered mostly novel rather than published variants [44].

The variants reported here are heterozygous, which is perhaps unsurprising as monogenic DNA repair conditions were not suspected clinically. Heterozygous variants of interest are reported in other sequencing studies of CVID cohorts [44, 45]. Genetic complexity in a predominantly sporadic condition like CVID could also be driven by non-coding regions, epistatic interactions, and incomplete penetrance. Functional validation of genetic candidates is further complicated by differing clinical presentations among family members with the same variant [46]. Careful selection of variants for functional follow-up in models incorporating multiple variants is needed for a complete picture of CVID pathogenesis.

We assessed differential expression of genes related to DNA repair in PBMCs from CVID patients and controls, anticipating subtle changes in expression. In comparing patients and controls, we observed increased expression of nucleotide excision repair factor, *ERCC2*. The gene product of *ERCC2*, XPD, is involved in transcription-coupled nucleotide excision repair, and increased expression has been associated with chemoresistance to cisplatin [47] and colorectal cancer [48]. No genes were found to be differentially expressed between infections-only CVID patients and controls. Complex CVID patients had decreased expression of *MSH6* and *TREX1* compared to both controls and infections-only CVID patients. MSH6 is a component of mismatch repair, and TREX1 is an intracellular exonuclease that degrades single- and double-stranded DNA in the cytoplasm [49] and is of interest in CVID given the elevated DNA damage and apoptosis we observe in this cohort. When comparing between CVID patient sub-groups, only those with a complex phenotype displayed increased expression of *TOP3A*, a DNA topoisomerase that controls the topology of DNA during replication and transcription. Increased mRNA expression of *TOP3A* is correlated with worse prognosis in non-small-cell lung cancer and lung adenocarcinomas [50].

A caveat to using PBMCs is cell type-specific effects may be diluted. Further analysis should focus on cell subsets given the documented perturbations in CVID B and T cell memory subset frequencies [51–55] and differences in methylation states in the naïve to memory B cell transition in CVID which affect transcriptional activity [56]. Additionally, some transcriptional changes may only become apparent upon stimulation.

A DNA repair defect was proven in some CVID patients with a homozygous stop codon in *NHEJ1* by measurement of γH2AX by microscopy and flow cytometry [57]. We found a greater induction of DNA damage following irradiation and delayed repair in a subset of patients, suggesting a failure of DNA repair machinery. The observed association of elevated T cell γH2AX expression and increasing age may be due to increased terminally differentiated effector T cells in CVID [58]. The controls included in this study are younger than patients due to local availability of donors. Further studies should feature more closely age-matched controls.

Following irradiation, a profound decrease in pATM was observed in a subset of CVID B cells. Cells with the lowest pATM levels also had decreased 53BP1 and higher γH2AX as compared to controls and other patients. The deficiency of pATM we observe in CVID B cells does not result in the neurodegenerative phenotype found in ataxia telangiectasia, so this is a less severe dysfunction and may reflect immune cell-specific deficiency. Recent evidence has implicated DDR pathways in inflammation [10, 59] and rheumatoid arthritis [60–63]. In rheumatoid arthritis T cells, reduced ATM was identified at baseline [63]. ATM deficiency prevented T cell proliferation and promoted premature apoptosis [60, 61]. A failure to induce pATM in the rheumatoid arthritis B cells 1 h post-irradiation was associated with skewed kappa light chain usage and an increased prevalence of CD21^lo^ B cells, a subset associated with CVID [64]. It would be of interest to study the role of ATM biology in CVID patients with greater proportions of CD21^lo^ B cells and according to Paris [55], Freiburg [54], and EUROClass [53] groups. Inflammatory processes can damage DNA [65–67]. While we cannot preclude that the DNA damage we observe is a result of inflammation rather than genetics, it is unlikely given that we do not observe differences between CVID patients with inflammatory complications and those without and samples were taken when disease was stable.

The low B cell numbers in some patients and the failure of naïve to memory transition may be due to increased susceptibility for apoptosis. Indeed, our previous RNAseq data [8] found elevated *FAS* gene expression in CVID B cells, and increased spontaneous apoptosis of CVID memory B cells has been shown in vitro [68, 69]. We measured the caspase-3 substrate, cleaved PARP, as an early marker of apoptosis at baseline and following irradiation. CVID T cells showed increased basal and induced levels of cleaved PARP, while CVID B cells had higher basal cleaved PARP, which further increased upon irradiation. These data are in accordance with other studies on apoptosis [69–72] and, given the association with decreased ATM, proposes deficient DNA repair as its mechanism.

## Conclusions

In conclusion, we identified rare and novel variants in genes related to DNA repair in a cohort of patients with sporadic CVID. The differential expression of genes *TOP3A*, *XRCC2*, and *MSH6* distinguishes patients with a complex CVID phenotype from those with an infections-only CVID phenotype and controls. Decreased pATM impairs the recruitment of other repair factors, delays damage repair and promotes apoptosis. Understanding the mechanism of antibody failure and malignancy risk in patients with complex CVID will aid clinical management and therapeutic development.

## Supplementary Information

Below is the link to the electronic supplementary material.

Supplementary file1 (DOCX 3605 KB)

Supplementary file2 (DOCX 102 KB)

## Data Availability

Upon request.

## Code Availability

Upon request.
