# Supplementary material for: Decreased ATM Function Causes Delayed DNA Repair and Apoptosis in Common Variable Immunodeficiency Disorders
Source: J Clin Immunol. 2021 May 19;41(6):1315–30. doi: 10.1007/s10875-021-01050-2 (PMC8310859; doi:10.1007/s10875-021-01050-2)
Supplement: Supplementary file 1 — Supplementary file1 (DOCX 3605 KB) [file 10875_2021_1050_MOESM1_ESM.docx]

**Electronic Supplementary Material: Figures**

**Decreased ATM function causes delayed DNA repair and apoptosis in common variable**

**immunodeficiency disorders**

**Authors:** Chantal E. Hargreaves^1*^, Silvia Salatino^2^, Sarah C. Sasson^1^, James E. G. Charlesworth^3^, Elizabeth Bateman^4^, Arzoo M. Patel^1^, Consuelo Anzilotti^5^, John Broxholme^2^, Julian C. Knight^2^, Smita Y. Patel^1, 5^

**Affiliations:**

^1^Nuffield Department of Medicine and Oxford NIHR Biomedical Research Centre, University of Oxford, Oxford, OX3 9DU, United Kingdom.

^2^Wellcome Centre for Human Genetics, University of Oxford, Oxford, OX3 7BN, United Kingdom.

^3^Oxford University Clinical Academic Graduate School, University of Oxford, Medical Sciences Office, John Radcliffe Hospital, Oxford, OX3 9DU, United Kingdom.

^4^Department of Immunology, Churchill Hospital, Oxford University Hospitals NHS Trust, Oxford, OX3 7LE, United Kingdom.

^5^Clinical Immunology Department, Oxford University Hospitals Trust, Oxford, OX3 9DU, United Kingdom.

*Corresponding author. Email: [chantal.hargreaves@ndm.ox.ac.uk](mailto:chantal.hargreaves@ndm.ox.ac.uk)

**Figure S1: Visualization of the overlap of variant-containing genes between patients.** The distribution of variant-containing genes (white nodes) amongst infections-only patients (red nodes) and complex patients (blue nodes).

**

**Figure S2: Frequencies of immune cell subsets as measured by TBNK assay at the time of blood sample collection for NanoString.** Frequency of immune cell subsets measured in CVID patients with infections-only (IO) and complex phenotypes (C). Each symbol represents an individual patient. Bar represents the median. Statistical significance was measured by 2-way ANOVA with Sidak’s multiple comparisons test.

****Figure S3: Correlation of immune cell frequencies and gene expression.** Associations between frequencies of immune cells, as measured by the TBNK assay, and NanoString gene expression log^2^ counts were determined by Spearman rank correlation. Each symbol represents an individual patient. Normal adult ranges are shaded in grey.

**Figure S4: Flow cytometric gating strategy of surface immune cell markers.**


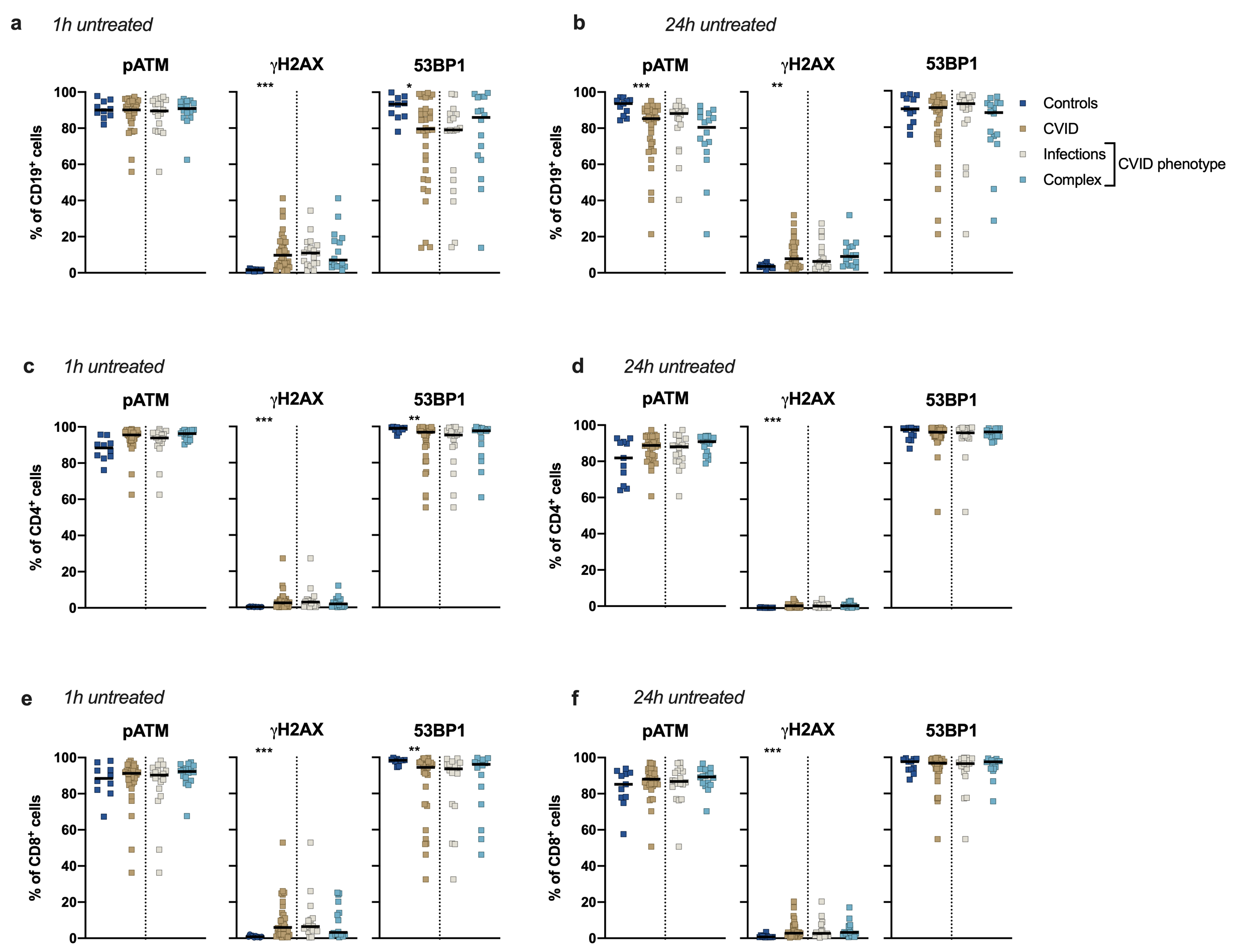


**Figure S5: CVID immune cell subsets have altered expression of DNA damage markers at baseline.** PBMCs from controls (n=11) and CVID and PAD patients (n=34 and n=2, respectively) were analysed by flow cytometry. Cells were in culture for 1 or 24 hours. The frequency of pATM+, H2AX+ and 53BP1+ cells amongst CD19+ B cells at (a) 1h and (b) 24h, CD4+ T cells at (c) 1h and (d) 24h and CD8+ T cells at (e) 1h and (f) 24h. Each symbol represents an individual control or patient. Bar represents the median. Statistical significance was determined by Mann-Whitney test.


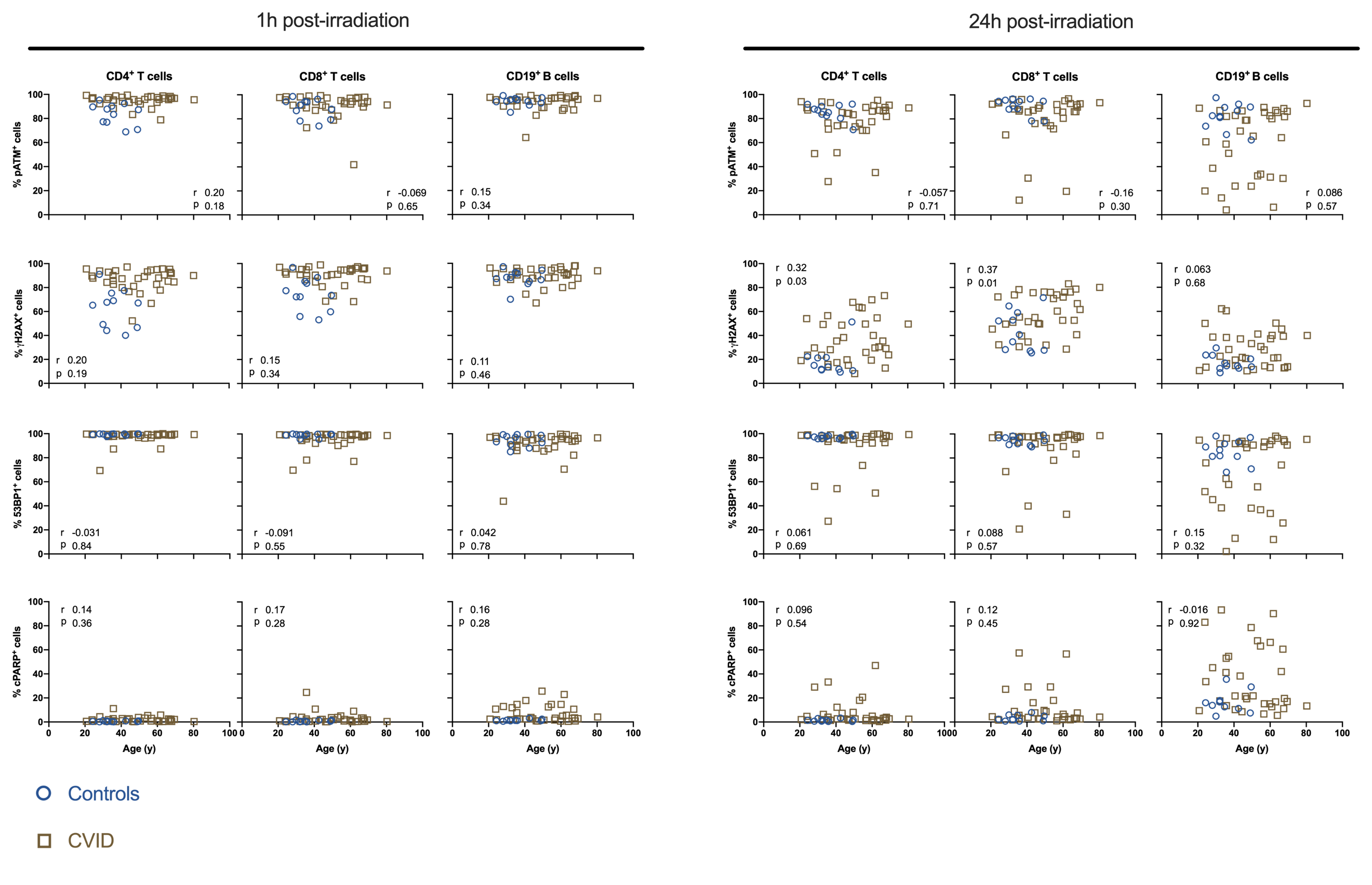


**Figure S6: Correlations of DNA damage markers with donor age at time of sampling.** Spearman rank correlations of the frequencies of immune cells expressing DNA damage markers with donor age (in years) at the time of blood draw. Displayed are controls (dark blue circles, n=11), CVID and PAD patients (gold squares, n=34 and n=2, respectively).

**Figure S7: Fold change in 53BP1 expression in controls and CVID patients.** Representative flow cytometric staining of 53BP1 and fold change in median fluorescence over untreated 1- and 24hs post-irradiation, respectively, in (a-b) CD19^+^ B cells, (C-D) CD4^+^ T cells and (e-f) CD8^+^ T cells in controls (blue circles) and CVID patients (gold squares)**.** Each symbol represents an individual. Bar represents the median. Statistical significance was measured by Mann-Whitney test.


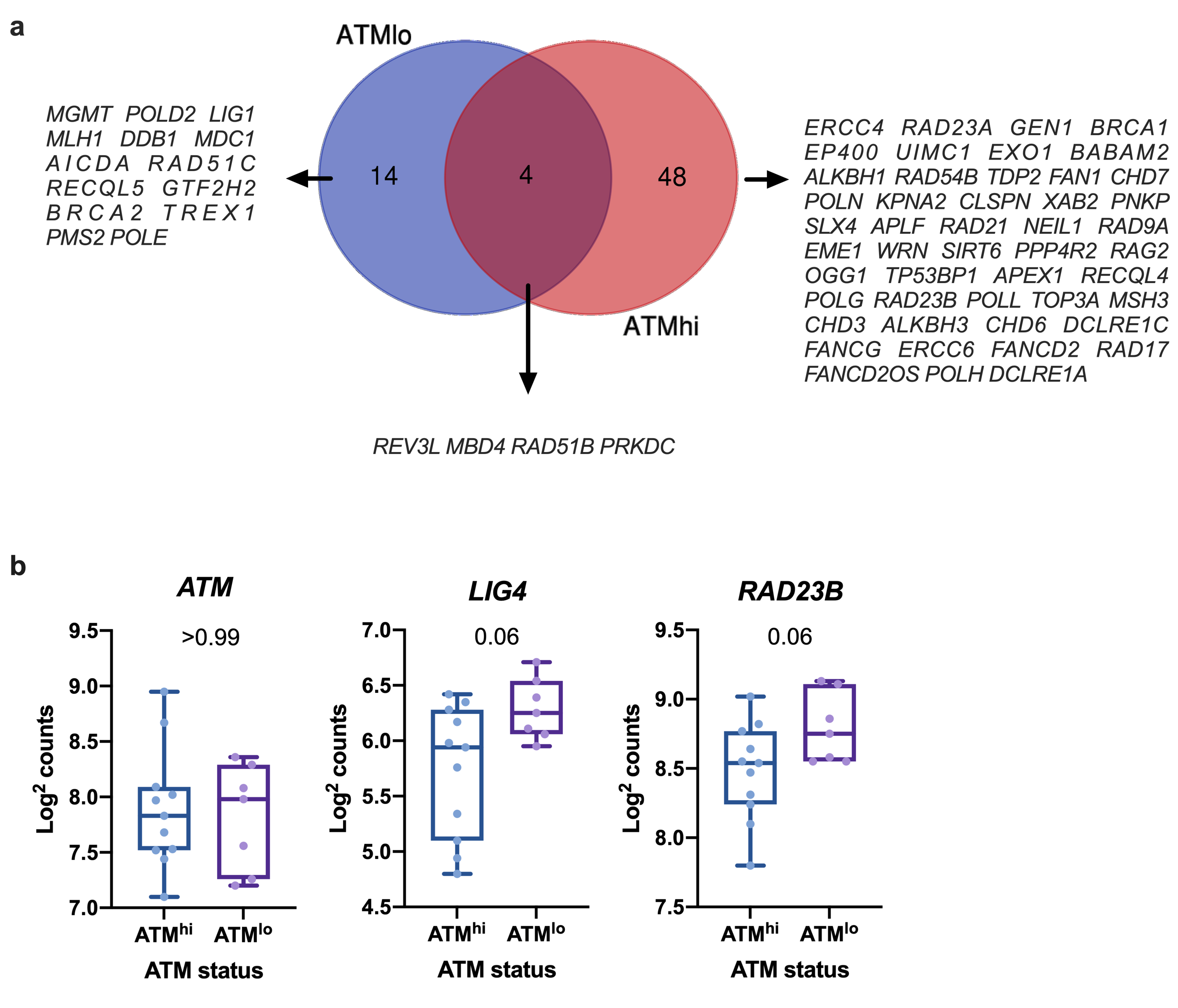


**Figure S8: Genetic signatures of ATM^lo^ status amongst CVID patients.** (a) Shared and distinct variable genes were compared between the two groups. (b) Expression of genes by Nanostring between the two groups.

**Figure S9: T cell apoptosis following DNA damage in controls and CVID patients.** Representative flow cytometric staining of cPARP and frequencies of cPARP^+^ cells in (a-b) CD4^+^ T cells and (c-d) CD8^+^ T cells in controls (blue circles) and CVID patients (gold squares)**.** Bar represents the median. Statistical significance was measured by Mann-Whitney test. The frequency of cPARP^+^ cells in controls (blue circles), ATM^hi^ patients (yellow diamonds) and ATM^lo^ patients (blue triangles) in (e) CD4^+^ and (f) CD8^+^ T cells. Statistical significance was measured by 2-way ANOVA with Sidak’s multiple comparisons test. Spearman rank correlation between the frequencies of (g) CD4^+^pATM^+^ and CD4^+^cPARP^+^ cells and (h) CD8^+^pATM^+^ and CD8^+^cPARP^+^ cells in controls (blue circles) and patients (gold squares). Each symbol represents an individual.
