# Supplementary material for: Decreased ATM Function Causes Delayed DNA Repair and Apoptosis in Common Variable Immunodeficiency Disorders
Source: J Clin Immunol. 2021 May 19;41(6):1315–30. doi: 10.1007/s10875-021-01050-2 (PMC8310859; doi:10.1007/s10875-021-01050-2)
Supplement: Supplementary file 2 — Supplementary file2 (DOCX 102 KB) [file 10875_2021_1050_MOESM2_ESM.docx]

**Electronic Supplementary Material: tables**

**Decreased ATM function causes delayed DNA repair and apoptosis in common variable**

**immunodeficiency disorders**

**Authors:** Chantal E. Hargreaves^1*^, Silvia Salatino^2^, Sarah C. Sasson^1^, James E. G. Charlesworth^3^, Elizabeth Bateman^4^, Arzoo M. Patel^1^, Consuelo Anzilotti^5^, John Broxholme^2^, Julian C. Knight^2^, Smita Y. Patel^1, 5^

**Affiliations:**

^1^Nuffield Department of Medicine and Oxford NIHR Biomedical Research Centre, University of Oxford, Oxford, OX3 9DU, United Kingdom.

^2^Wellcome Centre for Human Genetics, University of Oxford, Oxford, OX3 7BN, United Kingdom.

^3^Oxford University Clinical Academic Graduate School, University of Oxford, Medical Sciences Office, John Radcliffe Hospital, Oxford, OX3 9DU, United Kingdom.

^4^Department of Immunology, Churchill Hospital, Oxford University Hospitals NHS Trust, Oxford, OX3 7LE, United Kingdom.

^5^Clinical Immunology Department, Oxford University Hospitals Trust, Oxford, OX3 9DU, United Kingdom.

*Corresponding author. Email: [chantal.hargreaves@ndm.ox.ac.uk](mailto:chantal.hargreaves@ndm.ox.ac.uk)

**Table S1: Extended clinical characteristics of cohort**

| Sample ID | Sex | Age at onset (y) | Diagnosis | Clinical Phenotype | CD19 | CD3 | CD4 | CD8 | IgG | IgA | IgM |
| --- | --- | --- | --- | --- | --- | --- | --- | --- | --- | --- | --- |
|  |  |  |  | **Reference ranges:** | **0.1-0.5** | **0.7-2.1** | **0.3-1.4** | **0.2-0.9** | **6-16** | **0.8-3** | **0.4-2.5** |
| C099 | M | 31 | Complex | Thrombocytopenia | 0.16 | 1.8 | 0.59 | 0.88 | 8.89 | 0.13 ↓↓ | 0.34 ↓ |
| C166 | M | 64 | Complex | Organising pneumonia | 0.02 ↓↓ | 1.08 | 0.88 | 0.23 | 4.22 ↓ | 0.27 ↓↓ | 0.2 ↓ |
| C192 | M | 15 | Infections-only |  | 0.39 | 1.34 | 0.72 | 0.6 | 1.7 ↓↓ | 0.1 ↓↓ | 0.25 ↓ |
| D438 | F | 37 | Infections-only |  | 0.23 | 1.15 | 0.73 | n.d. | 4.08 ↓ | 0.2 ↓↓ | 0.38 ↓ |
| PID001 | M | 33 | Complex | Ulcerative colitis | 0.07 ↓ | 0.9 | 0.62 | 0.26 | 0.08 ↓↓ | 0.1 ↓↓ | 0.1 ↓↓ |
| PID002* | M | 34 | Complex | Polyclonal lymphoproliferation; lymphocytic interstitial pneumonitis; nodular regenerative hyperplasia of the liver; bronchiectasis | 0.14 | 0.94 | 0.54 | 0.36 | 0.35 ↓↓ | 0.07 ↓↓ | 0.16 ↓↓ |
| PID004* | F | 31 | Complex | Polyclonal lymphoproliferation; lymphocytic interstitial pneumonitis; autoimmune cytopenias; bronchiectasis | 0.14 | 1.31 | 0.68 | 0.48 | 3.35 ↓ | 0.44 ↓ | 0.17 ↓↓ |
| PID005 | F | 55 | Complex | Polyclonal lymphoproliferation; lymphocytic interstitial pneumonitis; nodular regenerative hyperplasia of the liver; splenomegaly | 0.64 ↑ | 0.98 | 0.71 | 0.27 | 0.32 ↓↓ | 0.08 ↓↓ | 0.26 ↓ |
| PID006 | M | 5 | Infections-only |  | 0.41 | 0.83 | 0.51 | 0.3 | 0.2 ↓↓ | 0.1 ↓↓ | 0.1 ↓↓ |
| PID008 | F | 31 | Infections-only | Bronchiectasis | 0.4 | 0.89 | 0.56 | 0.28 | 2 ↓↓ | 0.4 ↓ | 0.7 |
| PID010 | F | 30 | Infections-only |  | 0.26 | 1.18 | 0.82 | 0.43 | 2.6 ↓↓ | 0.1 ↓↓ | 0.16 ↓↓ |
| PID011* | F | 17 | Complex | Autoimmune cytopenias; splenomegaly; immune thrombocytopenia purpura; intermittent neutropenia | 0.11 | 0.63 ↓ | 0.52 | 0.12 | 9.81 | 0.09 ↓↓ | 0.08 ↓↓ |
| PID013 | F | 20 | Infections-only |  | 0.14 | 0.51 ↓ | 0.25 ↓ | 0.27 | 2 ↓↓ | 0.1 ↓↓ | 0.3 ↓ |
| PID016 | M | 30 | Infections-only |  | 0.18 | 1.75 | 0.76 | 0.9 | n.d. | n.d. | n.d. |
| PID017 | F | 33 | Infections-only |  | 0.2 | 1.66 | 0.59 | 0.87 | 0.2 ↓↓ | 0 ↓↓↓ | 0.2 ↓ |
| PID018* | F | 49 | Infections-only |  | 0.18 | 1.05 | 0.7 | 0.33 | 4.4 ↓ | 0.9 | 0.3 ↓ |
| PID019 | F | 17 | Primary antibody deficiency | Splenomegaly; lymphoid hyperplasia | 0.26 | 0.37 ↓ | 0.21 ↓ | 0.15 ↓↓ | 0.85 ↓↓ | 0.3 ↓↓ | 0.74 |
| PID020 | F | 37 | Infections-only | Coeliac disease | 0.34 | 0.86 | 0.54 | 0.27 | 3.07 ↓ | 0.06 ↓↓ | 0.07 ↓↓ |
| PID021* | M | 19 | Complex | Polyclonal lymphoproliferation; granulomatous interstitial lung disease; granulomatous inflammation lymph nodes; splenomegaly; lymphopenia; | 0.05 ↓ | 0.64 ↓ | 0.43 | 0.2 | n.d. | n.d. | n.d. |
| PID022 | F | 34 | Complex | Autoimmune cytopenias | 0.03 ↓↓ | 0.7 | 0.46 | 0.23 | 2 ↓↓ | 0.3 ↓↓ | 0.4 |
| PID027* | M | 5 | Infections-only |  | 0.5 | 3.8 ↑ | 2.2 ↑ | 1.2 ↑ | 2.7 ↓↓ | 0.3 ↓↓ | 0.4 |
| PID029* | M | 25 | Complex | Polyclonal lymphoproliferation; organising pneumonia; interstitial lung disease; | 0.18 | 0.69 ↓ | 0.41 | 0.25 | 2.63 ↓↓ | 0.15 ↓↓ | 0.27 ↓↓ |
| PID031* | F | 31 | Complex | Ulcerative colitis | 0.28 | 1.55 | 0.66 | 0.89 | 0.4 ↓↓ | 0.4 ↓ | 0.1 ↓↓ |
| PID032 | F | 11 | Complex | Autoimmune cytopenias | 0.04 ↓↓ | 1.6 | 0.76 | 0.76 | 1.2 ↓↓ | 0.1 ↓↓ | 0.045 ↓↓ |
| PID033* | M | 5 | Infections-only |  | 0.42 | 1.34 | 0.91 | 0.4 | 3 ↓ | 0.1 ↓↓ | 0.2 ↓ |
| PID034 | M | 3 | Infections-only |  | 0.2 | 1.15 | 0.45 | 0.69 | 2.6 ↓↓ | 0.1 ↓↓ | 0.1 ↓↓ |
| PID038 | M | 54 | Complex | Hypersensitivity pneumonitis; granulomatous interstitial lung disease | 0.26 | 1.51 | 0.42 | 1.09 ↑ | 0.79 ↓↓ | 0.06 ↓↓ | 0.05 ↓↓ |
| PID046 | F | 27 | Complex | Type 1 diabetes mellitus; hypothyroidism | 0.2 | 0.79 | 0.58 | 0.19 ↓ | 13.1 | 0.06 ↓↓ | 0.2 ↓ |
| PID047* | M | 13 | Infections-only |  | 0.44 | 1.05 | 0.68 | 0.37 | 2.7 ↓↓ | 0.2 ↓↓ | 0.5 |
| PID048* | F | 8 | Infections-only |  | 0.25 | 1.43 | 0.61 | 0.57 | 0.1 ↓↓ | 0.4 ↓ | 0.15 ↓↓ |
| PID049 | M | 35 | Complex | Bronchiectasis; thrombocytopenia; splenomegaly | 0.11 | 1.09 | 0.47 | 0.56 | 0.9 ↓↓ | 0.06 ↓↓ | 0.04 ↓↓ |
| PID050 | M | 13 | Complex | Lymphocytic colitis | 0.26 | 1.12 | 0.6 | 0.45 | 2.7 ↓↓ | <0.01 ↓↓ | 0.2 ↓ |
| PID054 | F | 17 | Infections-only |  | 0.04 ↓↓ | 1.64 | 0.85 | 0.83 | 2.3 ↓↓ | 0.1 ↓↓ | 0.2 ↓ |
| PID056 | M | 25 | Infections-only |  | 0.38 | 1.32 | 0.62 | 0.65 | 0.51 ↓↓ | 0.06 ↓↓ | 0.19 ↓↓ |
| PID057* | M | 14 | Infections-only |  | 0.06 ↓ | 0.6 ↓ | 0.39 | 0.22 | n.d. | n.d. | n.d. |
| PID064 | F | 19 | Complex | Enteropathy | 0.2 | 0.91 | 0.46 | 0.35 | 2.7 ↓↓ | 0.1 ↓↓ | 0.04 ↓↓ |
| PID068 | M | 4 | Primary antibody deficiency | Splenomegaly; thrombocytopenia; small volume lymphadenopathy | 0.22 | 1.88 | 1.19 | 0.69 | 10.4 | 0.06 ↓↓ | 0.05 ↓↓ |
| PID083 | M | 18 | Infections-only |  | 0.15 | 1.84 | 0.79 | 1.13 ↑ | 0.25 ↓↓ | 0.06 ↓↓ | 0.04 ↓↓ |
| PID093 | F | 48 | Infections-only |  | 0.2 | 1.14 | 0.62 | 0.47 | 2.1 ↓↓ | 0.07 ↓↓ | 0.7 |
| PID099 | F | 48 | Infections-only |  | 0.16 | 0.63 ↓ | 0.46 | 0.16 ↓ | 0.63 ↓↓ | 0.16 ↓↓ | 0.04 ↓↓ |

↓ = between lower normal and half lower normal values; ↓↓ = less than half lower normal value; ↓↓↓ = undetectable; ↑ = between upper normal value and twice upper normal value. The normal range is shown below the measured parameter. Values are at diagnosis unless otherwise stated. Not determined, n.d. An asterisk* denotes that the patient is in the ATM^lo^ flow cytometry group.

**Table S2: Table S6: Summary of experiment inclusion for patients and controls**

| Sample ID | HaloPlex (gDNA) | NanoString (PBMC lysate) | Flow cytometry (PBMCs) |
| --- | --- | --- | --- |
| HC007 |  |  | Yes |
| HC072 |  | Yes |  |
| HC074 |  | Yes | Yes |
| HC079 |  |  | Yes |
| HC080 |  | Yes | Yes |
| HC082 |  |  | Yes |
| HC084 |  | Yes | Yes |
| HC088 |  | Yes | Yes |
| HC089 |  |  | Yes |
| HC090 |  |  | Yes |
| HC092 |  | Yes | Yes |
| HC094 |  |  | Yes |
| HC102 |  | Yes |  |
| C099 | Yes |  |  |
| C166 | Yes |  |  |
| C192 | Yes |  |  |
| D438 | Yes |  |  |
| PID001 |  | Yes |  |
| PID002 | Yes |  | Yes |
| PID004 | Yes |  | Yes |
| PID005 | Yes |  | Yes |
| PID006 | Yes |  | Yes |
| PID008 | Yes | Yes | Yes |
| PID010 | Yes | Yes | Yes |
| PID011 | Yes | Yes | Yes |
| PID013 | Yes |  | Yes |
| PID016 | Yes | Yes | Yes |
| PID017 | Yes |  | Yes |
| PID018 | Yes |  | Yes |
| PID019 | Yes | Yes | Yes |
| PID020 | Yes |  | Yes |
| PID021 | Yes | Yes | Yes |
| PID022 | Yes |  | Yes |
| PID027 | Yes | Yes | Yes |
| PID029 | Yes | Yes | Yes |
| PID031 | Yes | Yes | Yes |
| PID032 | Yes | Yes | Yes |
| PID033 | Yes | Yes | Yes |
| PID034 | Yes |  | Yes |
| PID038 | Yes | Yes | Yes |
| PID046 | Yes | Yes | Yes |
| PID047 | Yes |  | Yes |
| PID048 | Yes |  | Yes |
| PID049 | Yes |  | Yes |
| PID050 |  | Yes |  |
| PID054 | Yes | Yes | Yes |
| PID056 | Yes | Yes | Yes |
| PID057 | Yes | Yes | Yes |
| PID064 | Yes | Yes | Yes |
| PID068 | Yes | Yes | Yes |
| PID083 | Yes |  | Yes |
| PID093 | Yes |  | Yes |
| PID099 | Yes |  | Yes |

**Table S3: HaloPlex HS assay design details and gene list.**

| Region Size : 1.810 Mbp |  |  |  |  |
| --- | --- | --- | --- | --- |
| Amplicon Summary | | | | |
| Total Amplicons | 79484 | | | |
| Total Target Bases Analyzable | 1.77 Mbp | | | |
| Total Sequenceable Design Size | 2.99 Mbp | | | |
| Target Coverage | 97.81% | | | |
| Target Parameters | | | | |
| Databases | RefSeq, Ensembl, CCDS, Gencode, VEGA, SNP, CytoBand | | | |
| Region | Coding Exons + UTRs + 5' UTR + 3' UTR | | | |
| Region Extension | 50 bases from 3' end and 50 bases from 5' end. | | | |
|  |  |  |  |  |
| Target ID | **Regions** | **% Coverage** | **% High Coverage (>=90%)** | **% Low Coverage (<90%)** |
| ACTR8 | 14 | 100 | 14 | 0 |
| AICDA | 4 | 95.59 | 4 | 0 |
| ALKBH1 | 7 | 100 | 7 | 0 |
| ALKBH2 | 3 | 100 | 3 | 0 |
| ALKBH3 | 12 | 99.72 | 12 | 0 |
| APEX1 | 3 | 96.09 | 3 | 0 |
| APEX2 | 6 | 100 | 6 | 0 |
| APITD1 | 7 | 98.11 | 7 | 0 |
| APLF | 19 | 99.33 | 19 | 0 |
| APTX | 12 | 100 | 12 | 0 |
| ASCC1 | 19 | 94.53 | 16 | 3 |
| ASCC3 | 42 | 98.02 | 41 | 1 |
| ATM | 61 | 96.81 | 55 | 6 |
| ATR | 46 | 98.92 | 44 | 2 |
| ATRIP | 15 | 100 | 15 | 0 |
| BABAM1 | 8 | 96.75 | 8 | 0 |
| BARD1 | 13 | 98.78 | 13 | 0 |
| BLM | 25 | 99.26 | 24 | 1 |
| BRCA1 | 25 | 98.31 | 24 | 1 |
| BRCA2 | 27 | 99.37 | 27 | 0 |
| BRCC3 | 12 | 98.29 | 12 | 0 |
| BRE | 17 | 99.98 | 17 | 0 |
| BRIP1 | 24 | 97.32 | 23 | 1 |
| C17ORF70 | 12 | 99.29 | 12 | 0 |
| C19ORF40 | 5 | 93.97 | 4 | 1 |
| C1ORF86 | 10 | 99.06 | 9 | 1 |
| CCNH | 9 | 99.68 | 9 | 0 |
| CDK7 | 12 | 98.4 | 11 | 1 |
| CDKN1A | 7 | 100 | 7 | 0 |
| CETN2 | 4 | 99.36 | 4 | 0 |
| CHD1L | 23 | 99.81 | 23 | 0 |
| CHD3 | 37 | 99.96 | 37 | 0 |
| CHD4 | 38 | 99.93 | 38 | 0 |
| CHD5 | 41 | 99.72 | 41 | 0 |
| CHD6 | 39 | 99.8 | 38 | 1 |
| CHD7 | 39 | 99.36 | 39 | 0 |
| CHD8 | 35 | 99.87 | 35 | 0 |
| CHEK1 | 13 | 99.28 | 13 | 0 |
| CHEK2 | 22 | 88.26 | 16 | 6 |
| chr12:1029831-1030031 | 1 | 35.32 | 0 | 1 |
| chr12:1064338-1064538 | 1 | 100 | 1 | 0 |
| chr12:8758710-8758910 | 1 | 100 | 1 | 0 |
| chr12:8762905-8763105 | 1 | 100 | 1 | 0 |
| chr12:8766261-8766461 | 1 | 100 | 1 | 0 |
| chr2:47646868-47647068 | 1 | 100 | 1 | 0 |
| chr2:47656701-47656901 | 1 | 88.56 | 0 | 1 |
| chr2:47663745-47663945 | 1 | 100 | 1 | 0 |
| chr2:47675231-47675431 | 1 | 92.04 | 1 | 0 |
| chr2:47680073-47680273 | 1 | 100 | 1 | 0 |
| chr2:47686473-47686673 | 1 | 81.59 | 0 | 1 |
| chr2:47692181-47692381 | 1 | 100 | 1 | 0 |
| chr5:131970785-131970985 | 1 | 100 | 1 | 0 |
| chr6:31717592-31717792 | 1 | 100 | 1 | 0 |
| chr6:31720933-31721133 | 1 | 100 | 1 | 0 |
| chr6:31725185-31725385 | 1 | 100 | 1 | 0 |
| chr6:31727374-31727574 | 1 | 100 | 1 | 0 |
| chr6:31729259-31729459 | 1 | 100 | 1 | 0 |
| chr7:6045534-6045734 | 1 | 94.03 | 1 | 0 |
| chr8:95388048-95388248 | 1 | 100 | 1 | 0 |
| chr8:95447805-95448005 | 1 | 100 | 1 | 0 |
| chr8:95459248-95459448 | 1 | 100 | 1 | 0 |
| chr8:95462595-95462795 | 1 | 91.04 | 1 | 0 |
| CLSPN | 27 | 99.74 | 27 | 0 |
| COPS2 | 14 | 98.26 | 12 | 2 |
| COPS3 | 13 | 98.7 | 12 | 1 |
| COPS4 | 13 | 97.7 | 12 | 1 |
| COPS5 | 11 | 94.77 | 9 | 2 |
| COPS6 | 3 | 96.31 | 3 | 0 |
| COPS7A | 10 | 99.83 | 10 | 0 |
| COPS7B | 10 | 98.6 | 10 | 0 |
| COPS8 | 10 | 100 | 10 | 0 |
| CUL4A | 21 | 99.45 | 21 | 0 |
| CUL4B | 25 | 99.09 | 25 | 0 |
| DCLRE1A | 9 | 99.95 | 9 | 0 |
| DCLRE1B | 4 | 100 | 4 | 0 |
| DCLRE1C | 20 | 99.03 | 20 | 0 |
| DDB1 | 21 | 98.01 | 20 | 1 |
| DDB2 | 9 | 99.93 | 9 | 0 |
| DEK | 12 | 99.26 | 12 | 0 |
| DNTT | 11 | 100 | 11 | 0 |
| ELL | 14 | 97.63 | 14 | 0 |
| EME1 | 5 | 98.69 | 5 | 0 |
| EME2 | 5 | 100 | 5 | 0 |
| EP400 | 51 | 100 | 51 | 0 |
| ERCC1 | 16 | 98.48 | 15 | 1 |
| ERCC2 | 15 | 99.38 | 15 | 0 |
| ERCC3 | 11 | 99.99 | 11 | 0 |
| ERCC4 | 13 | 95.06 | 11 | 2 |
| ERCC5 | 17 | 97.75 | 16 | 1 |
| ERCC6 | 24 | 99.75 | 24 | 0 |
| ERCC8 | 17 | 98.55 | 16 | 1 |
| EXO1 | 18 | 98.07 | 17 | 1 |
| FAM175A | 13 | 95.06 | 12 | 1 |
| FAN1 | 16 | 99.11 | 16 | 0 |
| FANCA | 36 | 97.53 | 34 | 2 |
| FANCB | 10 | 100 | 10 | 0 |
| FANCC | 21 | 99.8 | 21 | 0 |
| FANCD2 | 45 | 96.89 | 42 | 3 |
| FANCE | 10 | 98.79 | 10 | 0 |
| FANCF | 1 | 97.13 | 1 | 0 |
| FANCG | 11 | 99.81 | 11 | 0 |
| FANCI | 37 | 98.16 | 35 | 2 |
| FANCL | 14 | 100 | 14 | 0 |
| FANCM | 25 | 98.98 | 23 | 2 |
| FEN1 | 2 | 99.74 | 2 | 0 |
| GEN1 | 14 | 99.36 | 13 | 1 |
| GTF2H1 | 15 | 99.3 | 14 | 1 |
| GTF2H2 | 18 | 9.02 | 1 | 17 |
| GTF2H3 | 13 | 95.04 | 11 | 2 |
| GTF2H4 | 10 | 99.77 | 10 | 0 |
| GTF2H5 | 3 | 98.02 | 3 | 0 |
| H2AFX | 1 | 100 | 1 | 0 |
| HELLS | 23 | 98.1 | 23 | 0 |
| HELQ | 18 | 99.68 | 18 | 0 |
| HERC2 | 83 | 87.26 | 61 | 22 |
| HMGB1 | 7 | 89.94 | 6 | 1 |
| HMGB2 | 3 | 100 | 3 | 0 |
| HUS1 | 11 | 99.71 | 11 | 0 |
| INO80 | 37 | 98.73 | 34 | 3 |
| KAT5 | 5 | 99.35 | 5 | 0 |
| KIN | 13 | 93.55 | 12 | 1 |
| KPNA2 | 12 | 100 | 12 | 0 |
| LIG1 | 30 | 99.06 | 28 | 2 |
| LIG3 | 18 | 98.79 | 18 | 0 |
| LIG4 | 4 | 98.63 | 4 | 0 |
| MAD2L2 | 7 | 99.83 | 7 | 0 |
| MBD4 | 7 | 95.73 | 6 | 1 |
| MDC1 | 11 | 99.22 | 11 | 0 |
| MGMT | 9 | 98.43 | 8 | 1 |
| MLH1 | 24 | 100 | 24 | 0 |
| MLH3 | 15 | 97.85 | 13 | 2 |
| MMS19 | 25 | 99.73 | 24 | 1 |
| MNAT1 | 9 | 99.87 | 9 | 0 |
| MPG | 5 | 99.56 | 5 | 0 |
| MRE11A | 22 | 98.38 | 21 | 1 |
| MSH2 | 19 | 99.1 | 18 | 1 |
| MSH3 | 26 | 96.44 | 24 | 2 |
| MSH4 | 20 | 98.86 | 19 | 1 |
| MSH5 | 27 | 95.9 | 26 | 1 |
| MSH6 | 15 | 97.72 | 14 | 1 |
| MUS81 | 10 | 100 | 10 | 0 |
| MUTYH | 8 | 100 | 8 | 0 |
| NBN | 21 | 98.93 | 21 | 0 |
| NEIL1 | 9 | 98.34 | 9 | 0 |
| NEIL2 | 5 | 98.99 | 5 | 0 |
| NEIL3 | 10 | 95.83 | 8 | 2 |
| NHEJ1 | 13 | 100 | 13 | 0 |
| NTHL1 | 7 | 100 | 7 | 0 |
| NUDT1 | 6 | 98.62 | 6 | 0 |
| OGG1 | 9 | 98.72 | 9 | 0 |
| PALB2 | 15 | 99.84 | 15 | 0 |
| PARP1 | 24 | 100 | 24 | 0 |
| PARP2 | 12 | 100 | 12 | 0 |
| PARP3 | 10 | 100 | 10 | 0 |
| PAXIP1 | 22 | 98.08 | 21 | 1 |
| PCNA | 5 | 100 | 5 | 0 |
| PMS1 | 16 | 99.4 | 16 | 0 |
| PMS2 | 14 | 95.15 | 13 | 1 |
| PNKP | 7 | 100 | 7 | 0 |
| POLA1 | 37 | 99.33 | 36 | 1 |
| POLB | 16 | 97.47 | 15 | 1 |
| POLD1 | 22 | 99.7 | 22 | 0 |
| POLD2 | 9 | 100 | 9 | 0 |
| POLD3 | 16 | 100 | 16 | 0 |
| POLD4 | 2 | 96.29 | 2 | 0 |
| POLE | 37 | 98.82 | 36 | 1 |
| POLE2 | 20 | 96.81 | 17 | 3 |
| POLE3 | 2 | 100 | 2 | 0 |
| POLE4 | 6 | 99.49 | 6 | 0 |
| POLG | 22 | 99.77 | 22 | 0 |
| POLG2 | 12 | 99.57 | 12 | 0 |
| POLH | 11 | 85.12 | 10 | 1 |
| POLI | 14 | 99.24 | 14 | 0 |
| POLK | 17 | 98.02 | 16 | 1 |
| POLL | 7 | 99.63 | 7 | 0 |
| POLM | 9 | 100 | 9 | 0 |
| POLN | 31 | 98.87 | 31 | 0 |
| POLQ | 31 | 97.79 | 28 | 3 |
| POLR2A | 22 | 99.13 | 21 | 1 |
| POLR2B | 26 | 97.92 | 24 | 2 |
| POLR2C | 7 | 96.94 | 6 | 1 |
| POLR2D | 4 | 98.76 | 4 | 0 |
| POLR2E | 7 | 99.26 | 7 | 0 |
| POLR2F | 12 | 98.29 | 11 | 1 |
| POLR2G | 6 | 99.53 | 6 | 0 |
| POLR2H | 7 | 94.94 | 6 | 1 |
| POLR2I | 2 | 100 | 2 | 0 |
| POLR2J | 4 | 80.51 | 2 | 2 |
| POLR2K | 3 | 100 | 3 | 0 |
| POLR2L | 3 | 100 | 3 | 0 |
| PPP4C | 5 | 99.46 | 5 | 0 |
| PPP4R2 | 12 | 90.86 | 11 | 1 |
| PRKDC | 82 | 98.77 | 78 | 4 |
| RAD1 | 9 | 95.52 | 9 | 0 |
| RAD17 | 20 | 99.84 | 20 | 0 |
| RAD18 | 14 | 100 | 14 | 0 |
| RAD21 | 13 | 99.41 | 13 | 0 |
| RAD23A | 7 | 100 | 7 | 0 |
| RAD23B | 12 | 97.49 | 11 | 1 |
| RAD50 | 28 | 99.26 | 28 | 0 |
| RAD51 | 12 | 99.76 | 12 | 0 |
| RAD51B | 33 | 99.91 | 33 | 0 |
| RAD51C | 11 | 99.76 | 11 | 0 |
| RAD51D | 13 | 100 | 13 | 0 |
| RAD52 | 12 | 99.67 | 12 | 0 |
| RAD54B | 19 | 96.97 | 18 | 1 |
| RAD54L | 17 | 100 | 17 | 0 |
| RAD9A | 4 | 94.69 | 3 | 1 |
| RAD9B | 13 | 98.77 | 12 | 1 |
| RAG1 | 12 | 99.1 | 10 | 2 |
| RAG2 | 4 | 98.82 | 4 | 0 |
| RBBP8 | 27 | 98.7 | 26 | 1 |
| RBX1 | 5 | 100 | 5 | 0 |
| RDM1 | 6 | 100 | 6 | 0 |
| RECQL | 14 | 99.77 | 14 | 0 |
| RECQL4 | 7 | 99.13 | 7 | 0 |
| RECQL5 | 18 | 99.77 | 18 | 0 |
| REV1 | 24 | 99.84 | 24 | 0 |
| REV3L | 35 | 98.74 | 33 | 2 |
| RFC1 | 32 | 98.22 | 30 | 2 |
| RFC2 | 13 | 94.51 | 11 | 2 |
| RFC3 | 10 | 98.38 | 9 | 1 |
| RFC4 | 10 | 98.86 | 10 | 0 |
| RFC5 | 15 | 97.04 | 14 | 1 |
| RMI1 | 3 | 99.5 | 3 | 0 |
| RMI2 | 6 | 99.7 | 6 | 0 |
| RNF168 | 6 | 97.7 | 6 | 0 |
| RNF4 | 16 | 98.65 | 16 | 0 |
| RNF8 | 11 | 100 | 11 | 0 |
| RPA1 | 19 | 97.65 | 18 | 1 |
| RPA2 | 8 | 99.8 | 8 | 0 |
| RPA3 | 8 | 98.84 | 8 | 0 |
| RPA4 | 1 | 99.1 | 1 | 0 |
| SHFM1 | 15 | 97.9 | 13 | 2 |
| SIRT6 | 6 | 100 | 6 | 0 |
| SLX1A | 6 | 98.67 | 6 | 0 |
| SLX1B | 6 | 98.67 | 6 | 0 |
| SLX4 | 14 | 98.02 | 14 | 0 |
| SMUG1 | 8 | 99.51 | 8 | 0 |
| SPIDR | 32 | 97.27 | 30 | 2 |
| SPO11 | 14 | 98.93 | 13 | 1 |
| SPSB1 | 5 | 99.77 | 5 | 0 |
| STRA13 | 3 | 100 | 3 | 0 |
| TDG | 11 | 91.49 | 8 | 3 |
| TDP1 | 24 | 99.97 | 24 | 0 |
| TDP2 | 6 | 98.59 | 6 | 0 |
| TIMELESS | 21 | 99.57 | 21 | 0 |
| TLK1 | 29 | 96.88 | 28 | 1 |
| TOP2A | 33 | 98.05 | 32 | 1 |
| TOP2B | 36 | 96.86 | 33 | 3 |
| TOP3A | 21 | 96.63 | 18 | 3 |
| TOPBP1 | 27 | 98.3 | 25 | 2 |
| TP53 | 12 | 95.48 | 10 | 2 |
| TP53BP1 | 33 | 100 | 33 | 0 |
| TREX1 | 2 | 100 | 2 | 0 |
| TREX2 | 14 | 100 | 14 | 0 |
| UBE2T | 6 | 100 | 6 | 0 |
| UIMC1 | 20 | 95.97 | 19 | 1 |
| UNG | 8 | 99.97 | 8 | 0 |
| USP1 | 9 | 99.2 | 9 | 0 |
| USP45 | 23 | 98.18 | 21 | 2 |
| UVSSA | 14 | 99.43 | 13 | 1 |
| WHSC1 | 29 | 99.59 | 29 | 0 |
| WRN | 34 | 98.63 | 34 | 0 |
| XAB2 | 9 | 99.86 | 9 | 0 |
| XPA | 8 | 99.81 | 8 | 0 |
| XPC | 14 | 99.87 | 14 | 0 |
| XRCC1 | 15 | 99.9 | 15 | 0 |
| XRCC2 | 4 | 87.26 | 2 | 2 |
| XRCC3 | 14 | 98.88 | 14 | 0 |
| XRCC4 | 9 | 98.38 | 9 | 0 |
| XRCC5 | 23 | 99.58 | 22 | 1 |
| XRCC6 | 12 | 99.62 | 12 | 0 |
| YY1 | 5 | 99.88 | 5 | 0 |
| ZBTB32 | 8 | 100 | 8 | 0 |

| Filter | Variants (n) |
| --- | --- |
| Total | 84,922 |
| Pass QC | 46,343 |
| UK10K allele frequency <1% | 3,925 |
| 1000 Genomes project <1% | 2,459 |
| ESP6500 allele frequency <1% | 2,410 |
| ExAC allele frequency <1% | 2,243 |
| Include: transcript ablation, splice donor variant, splice acceptor variant, stop gained, frameshift variant, stop lost, initiator codon variant, transcript amplification, inframe insertion, inframe deletion, missense variant, splice region variant | 210 |
| Non-overlapping any low-complexity region annotated by RepeatMasker | 174 |
| Remove synonymous and non-coding variants | 136 |

**Table S4: HaloPlex HS filtering strategy.**

**Table S5: Variants of interest in genes related to DNA damage and repair pathways in Oxford sporadic CVID cohort**

| **Gene** | **dbSNP ID** | **Location** | **Reference Allele** | **Transcript Variant** | **Protein Variant** | **Consequence** | **ACMG** | **SIFT** | **PolyPhen** | **CADD** | **Mutation Assessor** | **FATHMM** | **M-CAP** | **GERP++** | **1000 Genomes** | **ExAC** | **gnomAD** | **Patients** |
| --- | --- | --- | --- | --- | --- | --- | --- | --- | --- | --- | --- | --- | --- | --- | --- | --- | --- | --- |
| *ALKBH3* | rs145265812 | 11:43905557 | C | c.208C>T | p.R70* | stop gained | Uncertain significance | - | - | 41 | - | - | - | 5.52 | 0.04 | 0.131 | 0.098 | PID099 |
| *ATM** | rs769346400 | 11:108158320 |  | c.3994-7C>T |  | splice region | Uncertain significance | - | - | - | - | - | - | - | - | - | 0.001 | PID049 |
| *CHD6* | rs190198493 | 20:40122175 |  | c.312+3A>G |  | splice region | Uncertain significance | - | - | - | - | - | - | - | 0.12 | 0.41 | 0.37 | PID049 |
| *CLSPN* | rs564344638 | 1:36230322 |  | c.134-8delT |  | splice region | Uncertain significance | - | - | - | - | - | - | - | 0.06 | - | 0.3 | PID002 |
| *GTF2H4* | rs752018406 | 6:30879930 |  | c.958+7C>T |  | splice region | Uncertain significance | - | - | - | - | - | - | - | - | 0.002 | 0.001 | PID016 |
| *HERC2* | rs375795824 | 15:28408233 |  | c.10746+7C>G |  | splice region | Uncertain significance | - | - | - | - | - | - | - | - | 0.02 | 0.015 | PID017 |
| *MSH3* | rs757194485 | 5:79974879 | AG | c.1310_1311delAG | p.E437fs*10 | frameshift | Uncertain significance | - | - | - | - | - | - | - | - | 0.002 | 0.003 | PID068 |
| *PARP2* | rs181717519 | 14:20824601 |  | c.1268+8T>G |  | splice region | Uncertain significance | - | - | - | - | - | - | - | 0.02 | 0.105 | 0.093 | PID099 |
| *POLH* | rs747834463 | 6:43581398 | CTC | c.1245-63_1245-61delCTC | p.P418del | inframe deletion | Uncertain significance | - | - | - | - | - | - | - | - | - | 0.06 | PID064 |
| *POLR2I* | rs116462592 | 19:36604975 |  | c.264-7C>A |  | splice region | Uncertain significance | - | - | - | - | - | - | - | - | - | 0.003 | C099 |
| *RAD21* | rs16889042 | 8:117879001 |  | c.-32-1G>A |  | splice acceptor | Uncertain significance | - | - | - | - | - | - | - | 0.08 | 0.13 | 0.119 | PID064 |
| *RAD52* | rs200674610 | 12:1038925 |  | c.348+60G>A |  | splice region | Uncertain significance | - | - | - | - | - | - | - | 0.14 | 0.12 | 0.12 | PID032 |
| *RAG1** | rs140212393 | 11:36587325 |  | c.-15+2277A>C |  | splice acceptor | Uncertain significance | - | - | - | - | - | - | - | 0.2 | - | 0.21 | PID064 |
| *TP53BP1* | rs540564502 | 15:43762077 | GGGATA | c.1347_1352delTATCCC | p.I450_P451del | inframe deletion | Uncertain significance | - | - | - | - | - | - | - | 0.18 | 0.61 | 0.61 | PID093 |
| *BRCA2* | rs398122570 | 13:32918775 | A | c.6922A>G | p.K2308E | missense | Uncertain significance | D | PrD | 27 | M | D | D | 4.95 | - | - | - | PID018 |
| *DCLRE1C** | rs41299658 | 10:14965056 | A | c.640T>A | p.L214M | missense | Uncertain significance | D | PrD | 25 | M | T | T | 5.47 | 0.04 | 0.094 | 0.07 | C192 |
| *DNTT* | rs142141382 | 10:98095667 | G | c.1379G>A | p.R460Q | missense | Uncertain significance | D | PrD | 33 | H | T | D | 5.96 | - | 0.007 | 0.006 | C099 |
| *EP400* | rs201361230 | 12:132549405 | G | c.8527G>A | p.V2843M | missense | Uncertain significance | D | PrD | 28 | L | D | D | 4.9 | - | 0.009 | 0.009 | PID038 |
| *KPNA2* | rs11545989 | 17:66038392 | C | c.494C>G | p.P165R | missense | Uncertain significance | D | PrD | 26 | H | T | D | 5.71 | 0.1 | 0.362 | 0.362 | C192 |
| *MBD4* | rs2307293 | 3:129150385 | C | c.1702G>C | p.D568H | missense | Uncertain significance | D | PrD | 25 | L | T | - | 5.12 | 0.42 | 0.539 | 0.532 | PID004 |
| *MLH3* | rs138006166 | 14:75485594 | C | c.4108G>A | p.A1370T | missense | Uncertain significance | D | PrD | 31 | M | T | D | 5.55 | - | 0.02 | 0.021 | PID083 |
| *MLH3* | rs376224686 | 14:75485639 | G | c.4063C>T | p.R1355C | missense | Uncertain significance | D | PrD | 25 | M | T | D | 5.7 | - | 0.003 | 0.004 | PID099 |
| *POLL* | rs200623399 | 10:103342588 | C | c.1126G>A | p.G376S | missense | Uncertain significance | D | PrD | 29 | H | T | D | 5.61 | - | 0.01 | 0.019 | PID032 |
| *POLL* | rs142726673 | 10:103343423 | G | c.907C>T | p.P303S | missense | Uncertain significance | D | PrD | 26 | M | T | D | 5.83 | 0.04 | 0.075 | 0.078 | PID032 |
| *POLM* | rs28382644 | 7:44118394 | C | c.659G>C | p.G220A | missense | Uncertain significance | D | PrD | 24 | - | T | - | 5.76 | 0.32 | 0.707 | 0.725 | C099 |
| *RAD23A* | rs769180661 | 19:13059619 | C | c.592C>G | p.L198V | missense | Uncertain significance | D | PrD | 24 | H | T | D | 4.61 | - | - | 0.001 | PID008 |
| *RNF168* | rs751363298 | 3:196198977 | G | c.1429C>G | p.R477G | missense | Uncertain significance | D | PrD | 23 | M | T | D | 6.08 | - | 0.002 | 0.002 | C166 |
| *TOP3A* | rs117856165 | 17:18205933 | C | c.604G>A | p.D202N | missense | Uncertain significance | D | PrD | 25 | M | . | - | 6.07 | 0.16 | 0.129 | 0.132 | PID099 |
| *ALKBH1* | rs747432161 | 14:78140453 | C | c.872G>T | p.R291L | missense | Uncertain significance | D | PoD | 27 | M | T | D | 5.95 | - | 0.002 | 0.001 | PID083 |
| *APEX1* | rs61757709 | 14:20924117 | A | c.103A>C | p.K35Q | missense | Uncertain significance | D | PoD | 26 | M | T | D | 5.81 | 0.02 | 0.016 | 0.019 | PID020 |
| *ERCC6* | rs41562713 | 10:50679030 | T | c.3061A>G | p.I1021V | missense | Uncertain significance | D | PoD | 25 | M | D | D | 5.8 | 0.02 | 0.105 | 0.106 | PID022 |
| *FANCD2* | rs765208098 | 3:10128861 | A | c.3379A>G | p.S1127G | missense | Uncertain significance | D | PoD | 26 | M | T | D | 5.54 | - | 0.001 | 0.001 | PID022 |
| *RECQL5* | rs369553617 | 17:73658785 | C | c.545G>A | p.R182H | missense | Uncertain significance | D | PoD | 24 | L | T | D | 5.54 | - | 0.006 | 0.006 | PID049 |
| *ATM** | rs1801673 | 11:108175463 | A | c.5558A>T | p.D1853V | missense | Uncertain significance | D | B | 24 | M | T | D | 5.52 | 0.18 | 0.516 | 0.496 | PID049 |
| *CHD3* | rs148625731 | 17:7814860 | G | c.5960G>A | p.R1987Q | missense | Uncertain significance | D | B | 24 | N | D | D | 4.21 | 0.02 | 0.017 | 0.017 | PID093 |
| *CHD6* | rs182761438 | 20:40126799 | T | c.1088A>T | p.Y363F | missense | Uncertain significance | D | B | 15 | - | D | - | 4.83 | 0.16 | 0.27 | 0.32 | PID020 |
| *FAN1* | rs375121625 | 15:31234088 | T | c.1919A>G; c.*323T>C | p.N640S | missense | Uncertain significance | D | B | 22 | N | T | T | 5.67 | - | 0.01 | 0.01 | PID006 |
| *FANCD2* | rs149125003 | 3:10088285 | T | c.1156T>G | p.F386V | missense | Uncertain significance | D | B | 16 | L | T | - | 5.73 | - | 0.34 | - | PID010 |
| *MMS19* | rs566453457 | 10:99238116 | C | c.293G>A | p.R98Q | missense | Uncertain significance | D | B | 25 | M | T | D | 5.99 | 0.12 | 0.03 | 0.021 | C099 |
| *POLG* | rs749355151 | 15:89860014 |  | c.3688T>G | p.S1230A | missense | Uncertain significance | D | B | 24 | L | D | D | 5.95 | - | 0.001 | 0.0004 | PID006 |
| *PRKDC* | rs201726098 | 8:48749063 | C | c.7787G>A | p.R2596H | missense | Uncertain significance | D | B | 23 | M | - | T | 5.67 | 0.02 | 0.011 | 0.013 | PID005 |
| *CHD6* | rs140007009 | 20:40079594 | G | c.3675C>G | p.H1225Q | missense | Uncertain significance | T | PoD | 23 | M | D | D | 5.78 | - | 0.094 | 0.091 | PID017 |
| *EXO1* | rs61750993 | 1:242022009 | G | c.745G>A | p.D249N | missense | Uncertain significance | T | PoD | 26 | L | T | T | 5.29 | 0.38 | 0.28 | 0.25 | PID093 |
| *KPNA2* | rs11545988 | 17:66039095 | C | c.646C>T | p.P216S | missense | Uncertain significance | T | PoD | 23 | N | T | - | 4.74 | 0.26 | 0.23 | 0.22 | C192; PID032 |
| *APEX2* | rs2301416 | X:55028863 | C | c.421C>T | p.R141C | missense | Uncertain significance | T | B | 16 | L | T | - | 5.65 | 0.08 | 0.31 | 0.32 | D438 |
| *PRKDC* |  | 8:48767861 | C | c.6683G>A | p.R2228K | missense | Uncertain significance | tolerated | benign | 23.3 | M | - | T | 5.06 | - | - | 0.0004 | PID032 |
| *RAD54B* |  | 8:95444390 |  | c.869G>A | p.R290Q | missense | Uncertain significance | tolerated | benign | 21.6 | L | T | D | 5.58 | - | - | 0.001 | PID017 |
| *APLF* | rs139869963 | 2:68729939 | A | c.245A>G | p.Y82C | missense | Uncertain significance | T | B | 16 | N | T | T | 5.22 | 0.18 | 0.36 | 0.36 | PID054 |
| *BRE* | rs148732163 | 2:28561330 | A | c.1102A>G | p.T368A | missense | Uncertain significance | T | B | 17 | N | - | - | 5.12 | 0.16 | 0.39 | 0.35 | PID005 |
| *FAAP100* | rs151231414 | 17:79517286 |  | c.1281A>G | p.R412G | missense | Uncertain significance | T | B | 11 | L | T | - | 4.58 | 0.48 | 0.53 | 0.5 | PID048; PID068 |
| *FAAP24* | rs149477674 | 19:33467502 |  | c.672A>G | p.S188G | missense | Uncertain significance | T | B | 14 | L | T | T | 5.35 | - | 0.01 | 0.01 | PID064 |
| *CHD6* | rs145769981 | 20:40081419 | G | c.3284C>T | p.A1095V | missense | Uncertain significance | T | B | 24 | N | T | T | 5.27 | 0.08 | 0.02 | 0.02 | PID020 |
| *DCLRE1A* | rs142138655 | 10:115609488 | G | c.1376C>T | p.P459L | missense | Uncertain significance | T | B | 13 | M | T | D | 5.63 | 0.02 | 0.02 | 0.03 | PID064 |
| *DCLRE1A* | rs61757204 | 10:115610043 | G | c.821C>G | p.A274G | missense | Uncertain significance | T | B | 11 | N | T | T | 6.02 | - | 0.07 | 0.07 | PID099 |
| *GEN1* | rs141482726 | 2:17952501 | C | c.749C>T | p.P250L | missense | Uncertain significance | T | B | 13 | L | T | T | 5.09 | 0.42 | 0.06 | 0.06 | PID005; PID008; PID010; PID022 |
| *GEN1* | rs17315702 | 2:17963171 | C | c.2692C>T | p.R898C | missense | Uncertain significance | T | B | 15 | N | T | - | 5.94 | - | 0.71 | 0.68 | C192 |
| *GTF2H2* | rs201330591 | 5:70351261 | A | c.388T>A | p.S130T | missense | Uncertain significance | T | B | 16 | N | T | T | 2.36 | 0.06 | 0.27 | 0.34 | PID002; PID018 |
| *MGMT* | rs143566017 | 10:131506256 | G | c.316G>A | p.A106T | missense | Uncertain significance | T | B | 10 | L | T | T | 4.79 | - | 0.002 | 0.002 | PID029 |
| *MSH6* | rs368318845 | 2:48025988 | G | c.866G>A | p.G289D | missense | Uncertain significance | T | B | 14 | L | D | D | 3.42 | - | 0.003 | 0.01 | C166 |
| *OGG1* | rs138037313 | 3:9792699 | G | c.208G>A | p.E70K | missense | Uncertain significance | T | B | 24 | M | T | T | 5.54 | 0.04 | 0.02 | 0.02 | PID054 |
| *PMS2* | rs138222146 | 7:6026780 | G | c.1616C>T | p.A539V | missense | Uncertain significance | T | B | 1 | L | T | T | 5.46 | - | 0.001 | 0.001 | PID002 |
| *POLD2* | rs149552381 | 7:44156484 | C | c.712G>A | p.V238I | missense | Uncertain significance | T | B | 14 | N | . | T | 5.77 | 0.02 | 0.02 | 0.02 | PID018 |
| *RAD17* | rs768109095 | 5:68682096 | A | c.788A>G | p.K263R | missense | Uncertain significance | T | B | 22 | L | T | T | 5.15 | - | 0.002 | 0.004 | PID006 |
| *RAD51B* | rs28908168 | 14:68353784 | G | c.619G>T | p.V207L | missense | Uncertain significance | T | B | 20 | N | T | T | 5.75 | 0.1 | 0.14 | 0.17 | PID018 |
| *SIRT6* | rs183444295 | 19:4174745 | C | c.856G>T | p.A286S | missense | Uncertain significance | T | B | 4 | L | T | T | 3.12 | 0.1 | 0.09 | 0.24 | PID083 |
| *SIRT6* | rs201141490 | 19:4174758 | G | c.843C>G | p.N281K | missense | Uncertain significance | T | B | 5 | L | T | D | 2.79 | 0.1 | 0.11 | 0.25 | PID083 |
| *TOP3A* | rs372360037 | 17:18181502 | G | c.2314C>G | p.L772V | missense | Uncertain significance | T | B | 14 | L | T | T | 5.55 | - | 0.005 | 0.005 | PID017 |
| *TOPBP1* | rs762407311 | 3:133368347 | C | c.1384G>A | p.A462T | missense | Uncertain significance | T | B | 5 | N | T | T | 5.57 | - | 0.002 | 0.001 | PID038 |
| *XAB2* | rs377180827 | 19:7692179 | A | c.472T>C | p.S158P | missense | Uncertain significance | T | B | 23 | M | T | D | 5.02 | - | - | 0.001 | PID020 |
| *POLR2E* | rs573559415 | 19:1088723 |  | c.*15-26_*15-5dupGTGTGACCACTCTCCCCCTTTC |  | splice region | Likely Benign | - | - | - | - | - | - | - | 0.64 | - | 1.478 | PID083 |
| *TP53BP1* | rs61751060 | 15:43769851 | A | c.880T>C | p.S294P | missense | Likely Benign | D | PrD | 24 | M | T | - | 5.52 | 0.32 | 0.641 | 0.634 | PID008 |
| *TREX1* | rs76493396 | 3:48506279 | G | c.2105G>A | p.R702Q; | missense | Likely Benign | D | PrD | 24 | L | T | - | 5.65 | 0.34 | 0.69 | 0.62 | PID027 |
| *BRCA1* | rs55650082 | 17:41245759 | C | c.1789G>A | p.E597K | missense | Likely Benign | T | PrD | 24 | M | D | D | 5.02 | - | 0.006 | 0.007 | PID013 |
| *BRIP1* | rs4988346 | 17:59924512 | C | c.577G>A | p.V193I | missense | Likely Benign | T | B | 0 | L | T | D | 5.26 | 0.14 | 0.4 | 0.37 | PID049; PID054 |
| *EP400* | rs117023015 | 12:132445421 | A | c.257A>G | p.N86S | missense | Likely Benign | T | B | 12 | L | D | - | 5.69 | 0.34 | 0.77 | 0.74 | PID004 |
| *MDC1* | rs2517560 | 6:30680968 | C | c.751G>A | p.E251K | missense | Likely Benign | T | B | 11 | N | T | - | 5.31 | 0.62 | 0.52 | 0.54 | PID002; PID027 |
| *PNKP* | rs34472250 | 19:50368466 | C | c.416G>A | p.R139H | missense | Likely Benign | T | B | 15 | M | T | T | 4.12 | 0.06 | 0.2 | 0.19 | PID022 |
| *POLD1* | rs3218775 | 19:50918229 |  | c.2546G>A | p.R849H | missense | Likely Benign | T | B | 22 | L | . | - | 4.58 | 0.46 | 0.67 | 0.79 | PID013 |
| *TDP2* | rs61757564 | 6:24658948 | T | c.92A>G | p.N31S | missense | Likely Benign | T | B | 9 | L | T | T | 5.65 | 0.04 | 0.21 | 0.19 | PID008 |
| *TP53BP1* | rs3803339 | 15:43724532 |  | c.3520A>G | p.I1174V | missense | Likely Benign | T | B | 9 | N | T | - | 4.79 | 0.94 | 0.89 | 0.93 | PID005 |
| *POLE* | rs61732929 | 12:133253974 | C | c.776G>A | p.R259H | missense | Likely Benign |  |  |  |  |  |  |  | 0 | 0 | 0 |  |
| *DCLRE1C* | rs148343292 | 10:14941186 | G | c.1282C>T | p.R428C | missense | Benign | - | B | 1 | - | T | - | 0.753 | 0.04 | 0.3 | 0.33 | PID017 |
| *UIMC1* | rs115224789 | 5:176335682 | A | c.1690T>C | p.Y564H | missense | Benign | D | PrD | 30 | M | T | T | 5.91 | 0.08 | 0.213 | 0.202 | PID020 |
| *EXO1* | rs4150001 | 1:242048680 | G | c.2276G>A | p.G759E | missense | Benign | D | B | 17 | L | T | - | 6 | 0.08 | 0.84 | 0.87 | PID010 |
| *WRN* | rs34477820 | 8:30916058 | A | c.95A>G | p.K32R | missense | Benign | D | B | 24 | M | T | T | 4.87 | 0.12 | 0.358 | 0.312 | PID016 |
| *RECQL4* | rs61754061 | 8:145739416 | C | c.1954G>A | p.V652M | missense | Benign | T | PoD | 20 | - | - | - | 5.37 | 0.04 | 0.22 | 0.24 | PID013 |
| *BRCA1* | rs28897689 | 17:41243509 | T | c.4039A>G | p.R1347G | missense | Benign | T | B | 22 | M | D | D | 5.19 | 0.06 | 0.4 | 0.38 | PID008 |
| *FANCD2* | rs145522204 | 3:10094159 | A | c.1634A>G | p.N545S | missense | Benign | T | B | 10 | N | T | T | 5.36 | 0.28 | 0.41 | 0.42 | C166 |
| *MLH1* | rs41294980 | 3:37067306 | G | c.1217G>A | p.S406N | missense | Benign | T | B | 13 | L | D | T | 5.8 | 0.04 | 0.09 | 0.09 | PID029 |
| *RAD51C* | rs147241704 | 17:56787304 | G | c.790G>A | p.G264S | missense | Benign | T | B | 23 | L | T | T | 6 | - | 0.19 | 0.18 | PID049 |
| *RAG2* | rs150762709 | 11:36615697 | C | c.22G>A | p.V8I | missense | Benign | T | B | 19 | L | D | D | 5.7 | 0.08 | 0.31 | 0.35 | PID016 |
| *SLX4* | rs111738042 | 16:3632347 | T | c.5501A>G | p.N1834S | missense | Benign | T | B | 8 | N | T | D | 5.62 | 0.2 | 0.54 | 0.56 | PID099 |
| *SLX4* | rs138615800 | 16:3656525 | C | c.710G>A | p.R237Q | missense | Benign | T | B | 0 | N | T | - | 5.16 | 0.6 | 0.5 | 0.47 | PID016 |

*Denotes known PID gene. Benign, B; damaging, D; high, H; low, L; medium, M, neutral, N; possibly damaging, PoD; probably damaging, PrD; tolerated, T.
